# Multiple Sporadic Colorectal Cancers Display a Unique Methylation Phenotype

**DOI:** 10.1371/journal.pone.0091033

**Published:** 2014-03-18

**Authors:** Victoria Gonzalo, Juan Jose Lozano, Virginia Alonso-Espinaco, Leticia Moreira, Jenifer Muñoz, Maria Pellisé, Sergi Castellví-Bel, Xavier Bessa, Montserrat Andreu, Rosa M. Xicola, Xavier Llor, Clara Ruiz-Ponte, Angel Carracedo, Rodrigo Jover, Antoni Castells, Francesc Balaguer, for the Gastrointestinal Oncology Group of the Spanish Gastroenterological Association

**Affiliations:** 1 Department of Gastroenterology, Hospital Clínic, Centro de Investigación Biomédica en Red de Enfermedades Hepáticas y Digestivas (CIBERehd), Institut d'Investigacions Biomediques August Pi i Sunyer (IDIBAPS), University of Barcelona, Barcelona, Catalonia, Spain; 2 Bioinformatics Unit, CIBERehd, Barcelona, Catalonia, Spain; 3 Department of Gastroenterology, Hospital del Mar, Barcelona, Catalonia, Spain; 4 Department of Medicine and Cancer Center, University of Illinois at Chicago, Chicago, Illinois, United States of America; 5 Galician Public Foundation of Genomic Medicine (FPGMX), CIBERER, Genomics Medicine Group, Hospital Clinico, Santiago de Compostela, University of Compostela, Galicia, Spain; 6 Gastroenterology Unit, Hospital General Universitario, Alicante, Spain; Sapporo Medical University, Japan

## Abstract

Epigenetics are thought to play a major role in the carcinogenesis of multiple sporadic colorectal cancers (CRC). Previous studies have suggested concordant DNA hypermethylation between tumor pairs. However, only a few methylation markers have been analyzed. This study was aimed at describing the epigenetic signature of multiple CRC using a genome-scale DNA methylation profiling. We analyzed 12 patients with synchronous CRC and 29 age-, sex-, and tumor location-paired patients with solitary tumors from the EPICOLON II cohort. DNA methylation profiling was performed using the Illumina Infinium HM27 DNA methylation assay. The most significant results were validated by Methylight. Tumors samples were also analyzed for the CpG Island Methylator Phenotype (CIMP); *KRAS* and *BRAF* mutations and mismatch repair deficiency status. Functional annotation clustering was performed. We identified 102 CpG sites that showed significant DNA hypermethylation in multiple tumors with respect to the solitary counterparts (difference in β value ≥0.1). Methylight assays validated the results for 4 selected genes (p = 0.0002). Eight out of 12(66.6%) multiple tumors were classified as CIMP-high, as compared to 5 out of 29(17.2%) solitary tumors (p = 0.004). Interestingly, 76 out of the 102 (74.5%) hypermethylated CpG sites found in multiple tumors were also seen in CIMP-high tumors. Functional analysis of hypermethylated genes found in multiple tumors showed enrichment of genes involved in different tumorigenic functions. In conclusion, multiple CRC are associated with a distinct methylation phenotype, with a close association between tumor multiplicity and CIMP-high. Our results may be important to unravel the underlying mechanism of tumor multiplicity.

## Introduction

Up to 10% of all colorectal cancer (CRC) patients develop more than one tumor in the colorectum, either synchronously (diagnosed at the same time) or metachronously (diagnosed during follow-up) [1], [2], [3]. Tumor multiplicity is thought to occur because of a common etiologic factor (genetic or environmental) and provide a good model to examine common molecular alterations and, more specifically, a potential field effect [4], [5], [6], [7]. Genetics explain only a part of the spectrum of multiple CRCs, especially those occurring in the context of Lynch syndrome (caused by mutations in the mismatch repair genes) [8], [9], [10], familial associated polyposis (FAP) [11], *MUTYH* associated polyposis (MAP) [11] and other forms of colorectal polyposis [12]. On the other side, the concept of field defect has been proposed to explain tumor multiplicity through a generalized cellular or molecular disorder in the entire colorectal mucosa, causing a putative field effect (so called “field cancerization”) [6], [7], such as in serrated polyposis syndrome [13], [14], [15]. However, the definitive underlying pathogenic mechanism of tumor multiplicity remains elusive.

In the non-hereditary scenario, previous studies have found common molecular alteration patterns between CRC pairs and in the normal colonic mucosa of patients with multiples colorectal tumors, supporting a putative field defect [4], [10], [14], [16]. In contrast to genetic alterations, which are not commonly found in normal mucosa from cancer patients, epigenetics are thought to play a major role in the carcinogenesis of those individuals that develop multiple tumors [4], [5], [14], [17], [18], [19], [20], [21]. In this sense, it has been suggested that synchronous CRCs are more frequently associated with the CpG island methylator phenotype (CIMP) [4], *BRAF* mutation and microsatellite instability [10]. Indeed, our group compared a set of 41 pair-wise multiple and solitary CRCs and identified hypermethylation of the *MGMT2* locus and *RASSF1A* gene as variables independently associated with tumor multiplicity. Moreover, several studies have found concordant methylation patterns in tumor pairs [4], [14], [17], [18]. On the other hand, global DNA hypomethylation has been linked to genomic instability and carcinogenesis [22], [23] and, recently, higher hypomethylation of LINE-1 (a surrogate marker of global DNA methylation) in normal colonic mucosa has been found to be a distinctive feature of patients with synchronous CRCs [14]. All these results suggest that shared environmental and/or genetic background may cause concordant patterns of DNA methylation in patients with multiple tumors. However, only a few methylation markers have been analyzed and high throughput techniques with genome wide capability are needed to find and better understand the underlying epigenetic signature of multiple sporadic CRCs.

In this study we aimed at describing the underlying epigenetic signature that differentiates multiple from solitary CRC tumors using a genome-wide approach. For this purpose, we analyzed 12 synchronous and 29 control solitary CRCs derived from the population-based EPICOLON-II cohort, and evaluated the genome-scale methylation profile using the Illumina Infinium HM27 DNA methylation assay, an approach that has not been previously attempted.

## Materials and Methods

### Patients and samples

Twelve patients with synchronous CRC and 29 age-, sex-, and tumor location-paired patients with solitary tumors were recruited from the EPICOLON II cohort, a multicenter population-based study performed in Spain between 2006 and 2007 [24]. Synchronous tumors were clearly separated by normal colonic mucosa and both were invasive (at least pT1). Patients were followed until death or March 2012, whichever came first. Demographic, clinical and tumor-related characteristics of patients included in the study are summarized in **Table 1**. Exclusion criteria for the present study were colorectal polyposis syndromes, Lynch syndrome, and personal history of inflammatory bowel disease. The Institutional Ethics Committee of each participating hospital (see Acknowledgements) approved the study, and written informed consent was obtained from all patients.

**Table 1:** Clinical and tumor characteristics of solitary and multiple colorectal cancer patients.

Clinico-pathological features	Solitary CRC patients (n = 29)	Multiple CRC patients (n = 12)	*p value*
**Age (years)**	71.1±9.1	74.0±7.1	0.33
**Age**			
<65years	6(20%)	1(8.3%)	0.65
≥65years	23(79%)	11(91.7%)	
**Gender**			
Male	20(69%)	9(75%)	1
Female	9(31%)	3(25%)	
**Body mass index (Kg/m** 2 **)**			
<30	23(82%)	9(75%)	0.67
≥30	5(18%)	3(25%)	
**Tumor location** 1			
Proximal	6 (20.6%)	2 (16.6%)	0.57
Distal	23 (79.4%)	10 (38.4%)	
**Family history of CRC in any first degree relative**			
No	26(89.6%)	8(66.7%)	0.91
Yes	3(10.3%)	4(33.3%)	
**Family history of Lynch-related tumor** * **in any first degree relative**			
No	22(75.9%)	8(66.7%)	0.39
Yes	7(24.1%)	4(33.3%)	
**Microsatellite instability status**			
Stable	25(86.2%)	12(100%)	0.4
Unstable	2(6.9%)	0(0%)	
**Tumor differentiation**			
Well or moderate	24(100%)^2^	11(100%)^3^	1
Poor	-	-	
**Mucinous tumor**			
No	20(83.3%)^2^	7(70%)^4^	0.394
Yes	4(16.7%)	3(30%)	
**TNM stage**			
I	4(13.8%)	2(16.7%)	0.298
II	9(31%)	6(50%)	
III	11(37.9%)	1(8.3%)	
IV	5(17.2%)	3(25%)	
**Somatic ** ***BRAF*** ** mutational status**			
Wild type	24 (100%)^2^	9 (100%)^5^	1
Mutated	-	-	
**Somatic ** ***KRAS*** ** mutational status**			
Wild type	14(58.3%)^2^	6(66.7%)^5^	1
Mutated	10(41.7%)	3(33.3%)	
**CIMP-high status** 6			
Positive	5(17.2%)	8(66.7%)	0.004
Negative	24(82.8%)	4(33.3%)	

* Lynch-related tumors: colorectal, endometrial, ovary, stomach, urinary tract, biliary, pancreas, brain.

1 Referred to the splenic flexure;

2 Referred to 24 patients;

3 Referred to 11 patients;

4 referred to 10 patients;

5 referred to 9 patients.

6 Based on Illumina Infinium DNA methylation assay.

CRC, colorectal cancer.

Frozen tumor colorectal tissues were obtained at surgery from all patients, and immediately stored at −80° until use. In patients with multiple lesions, tissue sample was obtained from one of the tumors (the most advanced or the largest one when multiple tumors had the same tumor stage).

### DNA extraction and bisulfite conversion

Frozen samples were thawed and genomic DNA was isolated using QIAamp DNA Mini Kit (Qiagen, Valencia, CA) according to the manufacturer's instructions. Bisulfite treatment was carried out on genomic DNA using the EZ DNA Methylation-Gold Kit (Zymo Research, Orange, CA) according to the manufacturer's protocol.

### Infinium array

We performed DNA methylation profiling from 12 synchronous and 29 solitary CRCs using Infinium methylation assay with HumanMethylation27 BeadChip (Illumina, San Diego, CA), which is capable of simultaneously analyze the methylation status of 27,578 individual CpG sites covering 14,495 protein-coding genes and 110 miRNAs [25], [26], [27]. Whole genome amplification, labeling, hybridization and scanning were performed according to the manufacturer's instruction at a core facility (Centre de Regulació Genòmica, Barcelona, Catalonia, Spain). Methylation status was measured as the ratio of signal from a methylated probe relative to both methylated and unmethylated probe signals. Methylation ratios were extracted using the Methylation Module in the Illumina Bead Studio following average normalization. Quantitative β-value ranges from 0 (0% methylation) to 1 (100% methylation). The p-value cut off for detected probes (different from background measurements) was set at 0.05. We excluded probes that were previously published to be unreliable (those containing single-nucleotide polymorphisms (SNPs) and those repetitive sequences that covered the targeted CpG dinucleotide) and those that were designed for sequences on either the X or the Y chromosome. Together, we masked data points for 7549 probes [27]. Complete microarray dataset is available at GEO (Gene Expression Omnibus; accession number GSE52573).

### Definition of CIMP-high tumors based on the Infinium assay

We classified tumors as CIMP-high (CIMP-H), CIMP-low (CIMP-L) and CIMP-0 based on a 2-step panels of markers recently described by Hinoue *et al* based on the Illumina Infinium HM27 DNA methylation assay [27]. The first panel (*B3GAT2*, *FOXL2*, *KCNK13*, *RAB31*, and *SLIT1*) qualifies a sample as CIMP (High and Low) versus CIMP-0 if β-value is ≥0.1 in three or more markers. The second marker panel (*FAM78A*, *FSTL1*, *KCNC1*, *MYODCD* and *SLC6A4*) distinguishes CIMP-H versus CIMP-L tumors if β-value is ≥0.1 in three or more markers (**Table 2**). These markers have shown to display 100% sensitivity and 100% specificity to identify CIMP-H tumors [27].

**Table 2:** Classification of solitary and multiple tumors according to the CIMP and *KRAS* status.

Tumor ID	*B3GAT2*	*FOXL2*	*SLIT1*	*RAB31*	*KCNK13*	CIMP-H or L (first panel)	Tumor ID	*FSTL1*	*FAM78A*	*MYOCD*	*SLC6A4*	*KCNC1*	CIMP-H (second panel)	Final CIMP classification	KRAS mutational status
**5628 (m)**	M	M	U	U	M	Yes	**5628 (m)**	M	U	M	U	M	Yes	**CIMP-H**	wild-type
**742 (m)**	M	M	U	U	M	Yes	**742 (m)**	M	U	M	M	U	Yes	**CIMP-H**	wild-type
**4147 (m)**	M	M	M	M	M	Yes	**4147 (m)**	U	M	M	M	M	Yes	**CIMP-H**	**mutated**
**3126 (m)**	M	M	M	M	M	Yes	**3126 (m)**	M	M	M	M	M	Yes	**CIMP-H**	**mutated**
**7742 (m)**	M	M	M	M	M	Yes	**7742 (m)**	U	M	M	U	M	Yes	**CIMP-H**	wild-type
**502 (m)**	M	M	M	M	M	Yes	**502 (m)**	M	M	M	M	M	Yes	**CIMP-H**	wild-type
**5063 (m)**	M	M	M	M	M	Yes	**5063 (m)**	M	M	M	U	M	Yes	**CIMP-H**	**mutated**
**2282 (m)**	M	M	U	U	M	Yes	**2282 (m)**	M	U	U	M	U	No	CIMP-L	NA
**5725 (m)**	U	M	U	U	M	No	**5725 (m)**	U	U	M	U	U	No	CIMP-0	wild-type
**4662 (m)**	M	M	M	M	M	Yes	**4662 (m)**	U	U	M	U	U	No	CIMP-L	wild-type
**5284 (m)**	M	M	U	U	M	Yes	**5284 (m)**	M	U	M	M	U	Yes	**CIMP-H**	NA
**5642 (m)**	M	M	M	U	M	Yes	**5642 (m)**	U	U	U	U	U	No	CIMP-L	wild-type
**5449 (s)**	M	M	U	M	M	Yes	**5449 (s)**	M	M	M	U	M	Yes	**CIMP-H**	wild-type
**5082 (s)**	M	M	M	M	M	Yes	**5082 (s)**	M	U	U	U	U	No	CIMP-L	**mutated**
**743 (s)**	M	M	M	U	M	Yes	**743 (s)**	U	U	U	U	M	No	CIMP-L	wild-type
**24058 (s)**	M	M	U	U	M	Yes	**24058 (s)**	U	U	U	U	M	No	CIMP-L	wild-type
**703 (s)**	M	M	M	U	M	Yes	**703 (s)**	M	U	U	U	M	No	CIMP-L	**mutated**
**2103 (s)**	M	M	U	M	M	Yes	**2103 (s)**	M	M	M	U	M	Yes	**CIMP-H**	wild-type
**13064 (s)**	M	M	M	M	M	Yes	**13064 (s)**	M	U	M	M	U	Yes	**CIMP-H**	wild-type
**13109 (s)**	M	M	M	U	M	Yes	**13109 (s)**	U	U	U	U	U	No	CIMP-L	**mutated**
**6068 (s)**	M	M	M	M	M	Yes	**6068 (s)**	M	U	U	U	U	No	CIMP-L	**mutated**
**1022 (s)**	U	M	U	U	M	No	**1022 (s)**	U	U	U	U	M	No	CIMP-0	**mutated**
**888 (s)**	M	M	M	M	M	Yes	**888 (s)**	M	U	U	U	U	No	CIMP-L	wild-type
**1562 (s)**	M	M	M	U	M	Yes	**1562 (s)**	U	U	M	U	U	No	CIMP-L	**mutated**
**5467 (s)**	M	U	M	U	M	Yes	**5467 (s)**	M	U	M	U	U	No	CIMP-L	wild-type
**887 (s)**	U	M	U	U	M	No	**887 (s)**	U	U	U	U	U	No	CIMP-0	wild-type
**5442 (s)**	M	M	M	M	M	Yes	**5442 (s)**	U	U	U	U	M	No	CIMP-L	**mutated**
**3226 (s)**	M	M	M	M	M	Yes	**3226 (s)**	M	U	U	U	U	No	CIMP-L	**mutated**
**7124 (s)**	M	M	U	U	M	Yes	**7124 (s)**	U	U	U	U	M	No	CIMP-L	wild-type
**3268 (s)**	M	M	U	U	M	Yes	**3268 (s)**	U	U	M	U	U	No	CIMP-L	**mutated**
**3183 (s)**	M	M	M	M	M	Yes	**3183 (s)**	M	M	M	U	M	Yes	**CIMP-H**	NA
**7564 (s)**	M	M	M	M	M	Yes	**7564 (s)**	M	M	M	M	M	Yes	**CIMP-H**	NA
**5085 (s)**	M	U	U	U	M	No	**5085 (s)**	U	U	M	U	U	No	CIMP-0	NA
**942 (s)**	M	M	M	U	U	Yes	**942 (s)**	M	U	M	U	U	No	CIMP-L	NA
**4146 (s)**	M	M	U	U	M	Yes	**4146 (s)**	U	U	U	U	U	No	CIMP-L	NA
**5616 (s)**	M	U	M	M	M	Yes	**5616 (s)**	M	U	M	U	U	No	CIMP-L	wild-type
**883A (s)**	U	M	U	U	M	No	**883A (s)**	U	U	U	U	U	No	CIMP-0	NA
**3383 (s)**	M	M	U	M	M	Yes	**3383 (s)**	U	U	M	U	U	No	CIMP-L	wild-type
**885 (s)**	M	M	M	U	M	Yes	**885 (s)**	M	M	U	U	U	No	CIMP-L	wild-type
**7422 (s)**	M	M	U	U	M	Yes	**7422 (s)**	U	U	U	U	U	No	CIMP-L	wild-type
**3267 (s)**	U	M	M	M	M	Yes	**3267 (s)**	M	M	U	U	U	No	CIMP-L	**mutated**

“(m)” indicate multiple tumors; “(s)” indicate solitary tumors; “M” indicates a β value of ≥0.1 (methylated); “U” indicates a β value of ≤0.1 (unmethylated); First panel classifies a tumor as CIMP-H or CIMP-L vs. CIMP-0; Second panel classifies a tumor as CIMP-H vs. CIMP-L/0; NA: not available.

### Technical validation of the Infinium assay using Methylight

Methylight technique for quantitative analysis of methylation was used for the technical validation of the results observed in the Illumina Infinium assay [28]. The following strict criteria were used to selected candidate genes for validation: 1) solitary tumor had a β value <0.2; and 2) multiple tumors had either a β value >0.3 and a difference in β value ≥0.2; and 3) adjusted p value <0.05; and 4) previous evidence of tumor suppressive features based on the published literature. Following these criteria, we selected 4 genes for technical validation (*MAP1B, HTRA1, ALOX15, TIMP3*). Locus specific PCR primers and probes are listed on **Table S1** and were specifically designed for bisulfited-converted DNA sequences and located at each gene promoter region. Methylight was carried out as previously described, using ALUC4 as internal control [17], [28].

### Evaluation of tumor mismatch repair deficiency

Tumor mismatch repair deficiency was evaluated by both microsatellite instability (MSI) testing and immunostaining including evaluation of MSH2, MLH1, MSH6 and PMS2 as previously described [29]. MSI status was assessed using BAT26 and NR24 quasimonomorphic markers as previously described [30]. Tumors were classified as MSI when either of the two markers was unstable.

### Evaluation of *BRAF* and *KRAS* mutation status

*BRAF* mutations at codon 600 in exon 15, and *KRAS* mutations at codons 12 and 13 in exon 2 were analyzed by Methylight and direct sequencing, respectively, as previously published [31].

### Functional annotation clustering of differentially methylated genes between multiple and solitary colorectal cancers

We used The Database for Annotation, Visualization and Integrated Discovery (DAVID) [32] to identify pathways relevant to carcinogenesis based on the genes that showed significantly differential methylation between multiple and solitary multiple tumors (difference in β value ≥0.1 and p<0.05) (DAVID: http://david.abcc.ncifcrf.gov).

### Statistical analysis

Logistic regression adjusted for age, sex and tumor location was used to evaluate the difference in DNA methylation β-values for each probe between two independent groups. The Illumina Infinium DNA methylation β-values were represented graphically using a heatmap, generated by the R/Bioconductor packages. Clinicopathological features were compared using Chi-square (qualitative variables) and t-tests (quantitative variables). Methylight quantitative data (percentage methylation ratio, PMR) was analyzed using the Mann-Whitney U test. A p-value<0.05 was considered statistically significant. Statistical analysis and data visualization were carried out using the R/Bioconductor software package and SSPS software (v.15).

## Results

### Differential methylation between multiple and solitary tumors

**Figure 1:**
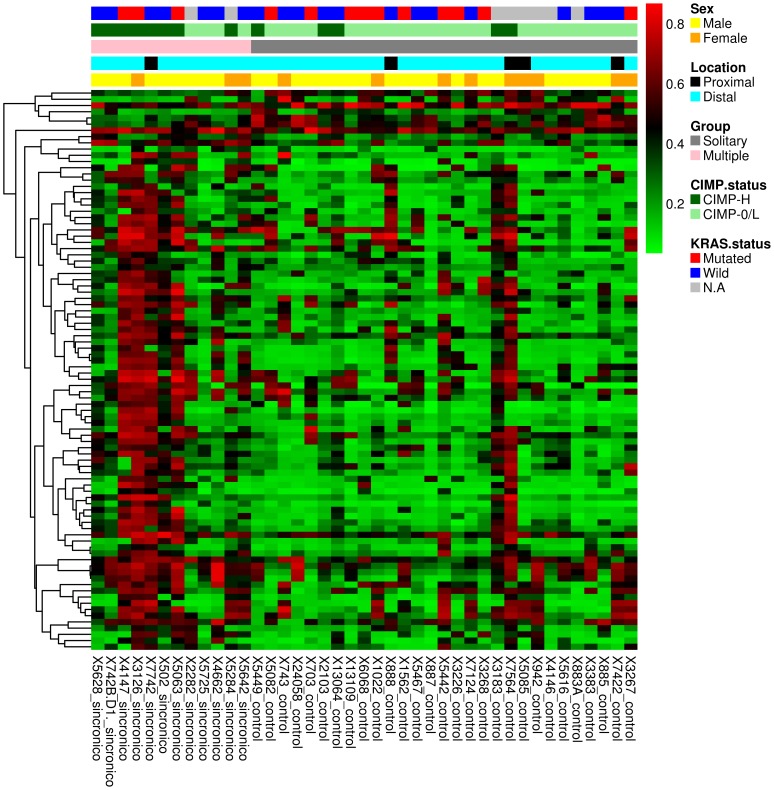
Heatmap showing the 90 most significantly hypermethylated CpG sites that differentiate multiple CRCs (n = 12) with respect to solitary tumors (n = 29) based on the Infinium DNA methylation data. The DNA methylation β-values are represented by using a color scale from red (high DNA methylation) to green (low DNA methylation). Rows represent probes and columns represent tumor samples. Clinical and molecular features (group, gender, tumor location, CIMP-H and *KRAS* mutational status) are represented above the heatmap with horizontal bars.

Twelve patients with multiple CRC and 29 age-, sex-, and tumor location-paired patients with solitary tumors constituted the basis of this study. Demographic and tumor characteristics from patients included in this study are listed in **Table 1**. We used Illumina Infinium HM27 DNA methylation assay, which assesses the DNA methylation status of 27,578 CpG sites located at the promoter regions of over 14,000 protein-coding genes. We identified 102 CpG sites that showed significant DNA hypermethylation in multiple tumors with respect to solitary ones (difference in β value ≥0.1 and p<0.05). Using more stringent criteria (difference in β value ≥0,2; p<0.05), we identified 36 CpG sites significantly hypermethylated (see detailed list of genes in **Table S2**). A heatmap showing the most significantly hypermethylated CpG sites that differentiate multiple and solitary tumors is shown in **Figure 1**. Overall, these results show that multiple tumors are associated with a distinct methylation phenotype, irrespective of age, sex and tumor location.

### Technical validation of microarray results

**Figure 2:**
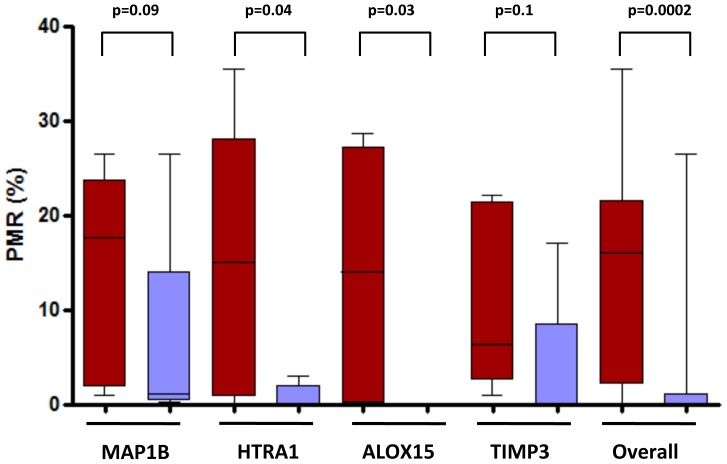
Technical validation of Infinium methylation data using Methylight assays. Four genes (*MAP1B, HTRA1, ALOX15, TIMP3*) were selected based on strict criteria (β value in solitary tumors <0.2; β value >0.3 in multiple tumors; difference in β value between multiple versus solitary ≥0.2; and an adjusted p value<0.05). Box-plots display the Percentage Methylation Ratio (PMR) determined by Methylight. The lines inside boxes denote median, and boxes mark the interval between the 25th and 75th percentiles. Black lines denote the highest and lowest PMR value. P values for the comparison between multiple (red) and solitary (blue) tumors (Mann-Whitney test) are shown.

In order to technically validate the results of Infinium assay we used stringent criteria to select probes that were significantly hypermethylated in multiple tumors compared to solitary lesions (β value in solitary tumors <0.2; β value >0.3 in multiple tumors; difference in β value between multiple and solitary tumors ≥0.2; and an adjusted p value<0.05). In order to select biologically relevant CpG sites, we prioritized genes with previous evidence of tumor suppressor features. Following these criteria, we selected *MAP1B*, *HTRA1*, *ALOX15*, and *TIMP3* for validation in five paired multiple and solitary tumors. Results are shown in **Figure 2**. Globally, we found a significantly higher methylation levels in multiple tumors compared to solitary ones (overall PMR, 14% versus 2.7%, respectively; p = 0.0002). As shown in **Figure 2**, all four markers showed higher levels of methylation in multiple tumors with respect to the solitary ones, thus reinforcing the consistency of our results.

### CIMP-high is associated with tumor multiplicity

**Figure 3:**
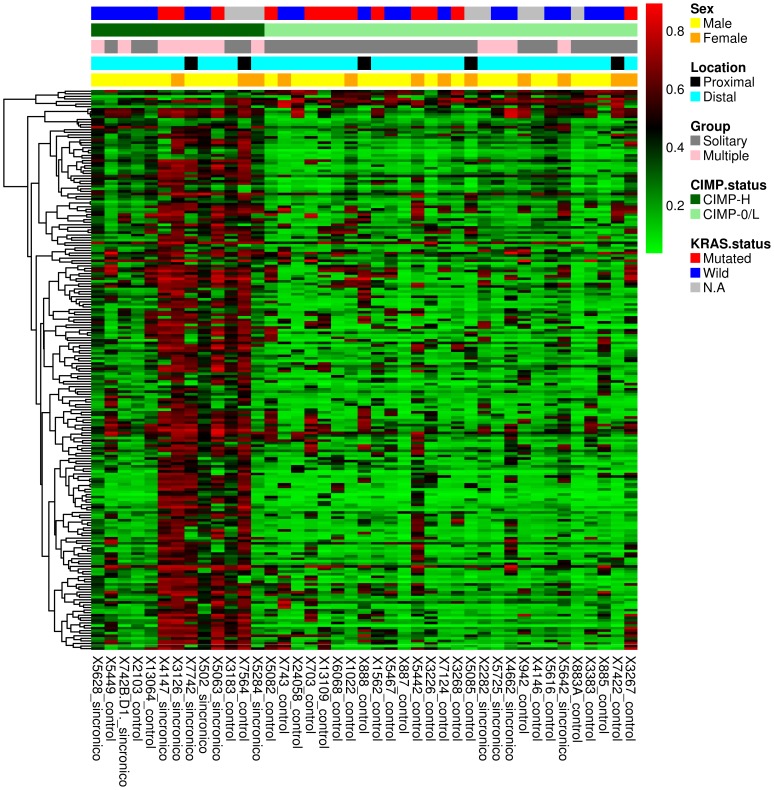
Heatmap showing the 218 most significantly hypermethylated CpG sites that differentiate CIMP-H (n = 13) and CIMP-0/L tumors (n = 28) based on the Infinium DNA methylation data. The DNA methylation β-values are represented by using a color scale from red (high DNA methylation) to green (low DNA methylation). Rows represent probes and columns represent tumor samples. Clinical and molecular features (group, gender, tumor location, CIMP-H and *KRAS* mutational status) are represented above the heatmap with horizontal bars.

**Figure 4:**
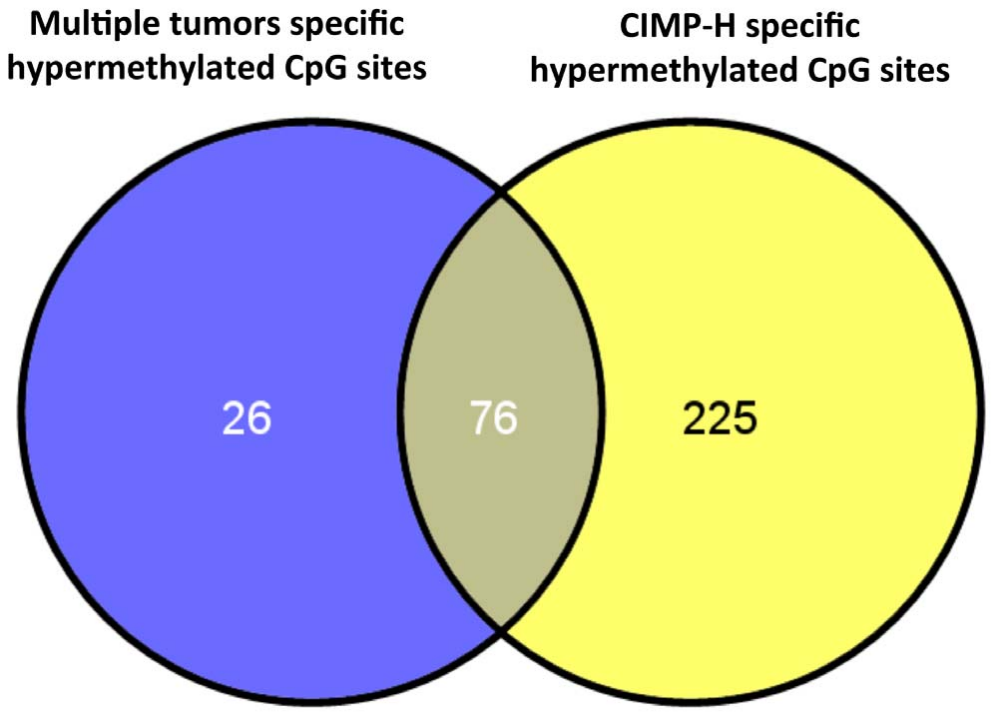
Overlap between significantly hypermethylated CpG sites in multiple and CIMP-H tumors. Blue circle shows 102 hypermethylated CpG sites found in multiple versus solitary tumors and yellow circle shows the 301 hypermethylated CpG sites in CIMP-H versus CIMP-L/0 tumors. Remarkably, 76 out of the 102 hypermethylated genes in multiple tumors were also seen to be hypermethylated in CIMP-H tumors, and are represented as an intersection.

We next analyzed the CIMP status of multiple and solitary tumors based on the recently developed gene marker panels defined by Hinoue *et al* [27]. This panel has recently shown to outperform the Methylight-based five-marker panel described by Weisenberger [33]. Ten out of the 12 (83%) multiple tumors and 25 out of the 29 (86.2%) solitary CRC showed hypermethylation of three or more markers from the first panel (i.e. *B3GAT2*, *FOXL2*, *KCNK13*, *RAB31*, and *SLIT1*), so they were classified as CIMP tumors. Based on the second panel (i.e. *FAM78A*, *FSTL1*, *KCNC1*, *MYOCD*, and *SLC6A4*), 8 out of the 12 (66.6%) multiple tumors were finally classified as CIMP-H, as compared to 5 out of the 29 (17.2%) solitary tumors (p = 0.004) (**Table 2**). CIMP-H tumors displayed significant hypermethylation (difference in β value ≥0.1; p value<0.05) in 301 CpG sites (109 with a difference in β value ≥0.2; p value<0.05). A heatmap showing the most significant CpG sites that differentiate CIMP-H and CIMP-L/0 tumors is shown in **Figure 3**. A detailed list with CIMP-H hypermethylated CpG sites is shown in **Table S3**. Interestingly, 76 out of the 102 hypermethylated CpG sites in multiple tumors were also seen to be hypermethylated in CIMP-H tumors (**Figure 4**). There were no *BRAF* mutations in any tumor. Our results show a close association between tumor multiplicity and CIMP, irrespective of age, sex and tumor location. This observation is in agreement with a previous larger study in which tumors were classified using Methylight-based markers [4], thus reinforcing the field-defect theory.

### Association between *KRAS* mutations and hypermethylation

*KRAS* mutations have been associated to a methylation phenotype called CIMP-low, in which hypermethylation of a reduced number of CIMP-defining loci occur [27]. We sought to investigate the methylation profile associated with *KRAS* mutant tumors and its association with tumor multiplicity. We found that *KRAS* mutant tumors were represented in both multiple and solitary tumors (33.3% versus 43.4%, respectively; p = 0.7) (**Figure 1**). Interestingly, we found that *KRAS* mutant tumors showed a distinct methylation profile compared to *KRAS* wild-type tumors. We identified 189 CpG sites that showed significant DNA hypermethylation in *KRAS* mutant CRCs with respect to *KRAS* wild-type tumors (difference in β value ≥0.1 and p<0.05). Using more stringent criteria (difference in β value ≥0,2; p<0.05), we identified 92 CpG sites significantly hypermethylated. A detailed list with *KRAS*-associated hypermethylated CpG sites is shown in **Table S4 and Figure S1**. The percentage of CIMP-H did not differ between *KRAS* mutant and wild-type tumors (23% versus 35%, respectively; p = 0.7). Similarly, the percentage of CIMP-low did not differ between *KRAS* mutant and wild-type tumors (69.2% versus 55%, respectively; p = 0.485). Overall, although we found that *KRAS* mutated tumors display a distinct methylation profiles, there was association with neither tumor multiplicity nor CIMP status.

### Functional analysis of differential methylation observed in multiple colorectal cancer

We performed a enrichment analysis on the 102 hypermethylated probes observed in multiple tumors (β value >0,1; p<0.05) using the Database for Annotation, Visualization and Integrated Discovery tool in order to find a functional correlation in any carcinogenic pathway involved in carcinogenesis. This functional analysis showed the presence and enrichment of genes involved in different tumorigenic functions: cell motion (12 genes), cell migration (7 genes), pathways in cancer (8 genes), cell motility (7 genes), regulation of cell proliferation (11 genes), transcription factor activity (14 genes), and transcription regulation (17 genes) (**Table 3**). Full list of functional annotation clustering of differentially methylated genes is shown in **Table S5**.

**Table 3:** Functional annotation clustering of differentially methylated genes found in multiple versus solitary tumors based on DAVID analysis.

Category	Term	Count	P value	Genes
GOTERM_BP_FAT	GO:0006928∼cell motion	12	1.66821E-05	*FGF19, SMO, RET, GDF7, ARHGEF7, UNC5A, ERBB2, GBX2, DPYSL5, KITLG, CXCL12, RUNX3*
GOTERM_MF_FAT	GO:0003700∼transcription factor activity	14	0.000934574	*IRX3, THRB, SOX14, SOX5, ZNF232, SOX8, GLI3, DLX5, GBX2, HIF3A, TFAP2A, ALX4, RUNX3, FOXE3*
GOTERM_BP_FAT	GO:0016477∼cell migration	7	0.002226607	*FGF19, SMO, RET, ARHGEF7, GBX2, KITLG, CXCL12*
KEGG_PATHWAY	hsa05200:Pathways in cancer	8	0.002975368	*FGF19, SMO, RET, ERBB2, WNT9B, KITLG, GLI3, DAPK1*
GOTERM_BP_FAT	GO:0048870∼cell motility	7	0.003771569	*FGF19, SMO, RET, ARHGEF7, GBX2, KITLG, CXCL12*
GOTERM_BP_FAT	GO:0042127∼regulation of cell proliferation	11	0.004572205	*SMO, HRH3, CCKBR, ERBB2, DLX5, KITLG, PDGFC, IGFBP3, GLI3, FOXE3, RUNX3*
SP_PIR_KEYWORDS	transcription regulation	17	0.009600471	*IRX3, MTERF, ZNF264, THRB, SOX14, SOX5, ZNF232, PRDM16, SOX8, GLI3, ZNF681, GBX2, HIF3A, TFAP2A, ALX4, RUNX3, FOXE3*

## Discussion

In this study we examined for the first time the genome-scale DNA methylation profile of tumor tissues from patients with multiple and solitary CRC recruited from a population-based cohort. We found that tumor multiplicity is associated with a distinct methylation profile, regardless of age, sex or tumor location. Compared with solitary tumors, multiple CRCs showed significant hypermethylation at specific CpG sites and, interestingly, there was a strong association with the CIMP-H described for CRC. Functional analysis of differentially methylated CpG sites in multiple tumors showed enrichment of genes involved in different tumorigenic functions. Results from the methylation profiling were successfully validated by quantitative PCR assays. Overall, our data provide new insight into the field cancerization effect and colorectal carcinogenesis in non-hereditary cases. This study reveals that somatic hypermethylation plays an important role in tumor multiplicity and may constitute an interesting biomarker for CRC risk assessment.

Recent studies have reported a close association between aberrant DNA methylation and tumor multiplicity [4], [14], [16], [17], [18]. Nosho and colleagues [4] analyzed 47 patients with synchronous CRC and 2021 solitary tumors for several methylation markers, including 8 CIMP-specific CpG island (i.e. *CACNA1G*, *CDKN2A*, *CRABP1*, *IGF2*, *MLH1*, *NEUROG1*, *RUNX3*, and *SOCS1*) and found a significant association between tumor multiplicity and the presence of CIMP-high (35% in synchronous tumors versus 8% in solitary tumors; p = 0.036). More importantly, the authors found concordant methylation within tumor pairs. Similarly, Konishi and colleagues [18] analyzed the methylation status of a limited number of makers in 57 multiple tumors and 69 solitary CRCs, and found that the methylation status of *p14* and *MGMT* was significantly higher in multiple tumors, showing concordant methylation for some markers within tumors pairs of the same colonic site. In line with these observations, we previously showed that hypermethylation of *MGMT* and *RASSF1A* is independently associated with tumor multiplicity [17]. In another study, Kamiyama and colleagues [14] analyzed the methylation status of long interspersed nucleotide element-1 (LINE-1) in matched cancer tissue and non-cancerous colonic mucosa from patients with single and multiple right-sided CRCs. The authors found higher hypomethylation of LINE-1 in both tumor and normal mucosa from patients with multiple tumors compared to patients with solitary tumors, and more importantly, LINE-1 hypomethylation was an independent predictor for metachronous tumors (p = 0.003). The authors suggested that LINE-1 hypomethylation in normal mucosa could be used as an epigenetic predictive biomarker for multiple CRC risk. It is important to note that LINE-1 hypomethylation has been previously found to be inversely correlated with the CIMP phenotype, which may be in contradiction with our and previous studies. However, the correlation between LINE-1 hypomethylation and CIMP in multiple tumors has not been explored in depth, and differences in patient selection and methodology could explain these unexpected results. Finally, other studies have hypothesized that the genetic and epigenetic landscape of a given tumor is determined by the location in the colon, and that similar molecular profiles for synchronous tumors is influenced by proximity [34], [35]. Unfortunately, we could not subanalyze this issue due to the unavailability of the second neoplasm. All these results suggest that accumulation of aberrant DNA methylation occurs predominantly in individuals with a propensity to develop multiple tumors. The results of the present study not only argue in favor of this hypothesis, but also provide new evidence about the epigenetic landscape of patients with multiple tumors. The underlying mechanism of the association between aberrant methylation and multiplicity is still unknown. Some authors have suggested an inherited predisposition in some cases [14], with the accumulation of methylation errors during aging in a genetically predisposed subgroup of individuals. However, this hypothesis remains unproven and future studies are needed.

In this study we successfully validated by Methylight the methylation status of 4 differentially methylated CpG sites observed in the discovery phase of the study. Specifically, we observed that *MAPB1B*, *HTRA1*, *ALOX15*, and *TIMP3* were significantly hypermethylated in multiple tumors. *MAP1B* (Microtubule-Associated Protein 1B) has been previously shown to be hypermethylated in CIMP-high tumors without MSI, which mainly correspond to the group of tumors analyzed in our study [36]. *HTRA1* is a member of the HTRA (High-Temperature Requirement Factor A) family of serine proteases and plays a protective role in various malignancies due to its tumor suppressive properties [37], [38], [39]. *HTRA1* has shown to be silenced through promoter hypermethylation [38], and proposed as a potential novel biomarker for diagnosis and prediction in several cancers. *ALOX15* (15-lipoxygenase or 15-LOX) is an inducible and highly regulated enzyme in normal human cells that plays a key role in the production of lipid signaling mediators. *ALOX15* has recently shown to be down-regulated in CRC and act as a tumor suppressor by promoting various anti-tumorigenic events, including cell differentiation and apoptosis, and inhibits chronic inflammation, angiogenesis and metastasis [40]. Finally, Tissue Inhibitor of Metalloproteinases-3 (*TIMP-3*) has found to be silenced in several types of cancer by promoter gene hypermethylation, including CRC [41], [42]. Overall, our results show that multiple tumors are associated with hypermethylation of well-established tumor suppressor genes.

Independently of the underlying mechanism behind the strong association between aberrant methylation and tumor multiplicity, our results suggest that the methylation status of specific markers could be used to stratify the risk of tumor multiplicity. Kamiyama and colleagues recently showed that LINE-1 methylation status in normal colonic mucosa could predict the development of metachronous CRC with high sensitivity [14], thus representing a clinically important prognostic biomarker for the identification of “high-risk” patients. Similarly, the analysis of the methylation status of specific markers identified in our study could be used in a clinical scenario to identify high-risk patients and tailor the surveillance strategy. Prospective studies specifically analyzing this hypothesis, however, are warranted.

The main strength of this study is that we utilized a population-based cohort of well-described CRC cases, thus minimizing the selection bias. Moreover, we used for the first time genome-wide methylation profiling with Illumina Infinium assay in this setting. However, we are aware of some limitations. First, we did not analyze DNA methylation correlation in tumor pairs due to the design of the EPICOLON II project, in which only one tumor was collected. Second, CIMP definition was not based on previously described methylation markers [33]. However, there is currently no consensus definition of CIMP tumors, and Hinoue and colleagues [27] recently showed that a new panel based on the Illumina Infinium DNA methylation platform outperformed the Methylight-based five-marker panel (i.e. *CACNA1G*, *IGF2*, *NEUROG1*, *RUNX3* and *SOCS1*). The frequency of CIMP-high frequency in solitary CRCs observed in our study (17%) is in line with previous figures, which reinforces the accuracy of the new panel proposed by Hinoue *et al.* Third, in our study, there were not *BRAF* mutant tumors, and accordingly, the association of tumor multiplicity with a distinct methylation phenotype refers only to CIMP-high/*BRAF* wild-type tumors, which can represent up to 40% of CIMP-high tumors. Finally, as our results should be formally considered not statistically significant when applying multiple testing corrections, additional studies in other cohorts are needed in order to confirm the results. However, we were able to confirm some of the most significant hypermethylated CpG sites by Methylight, thus reinforcing the validity of our results.

In summary, our results are consistent with the hypothesis that tumor multiplicity is associated with a distinct pattern of aberrant methylation. Compared with solitary tumors, multiple CRCs display more frequently CIMP-H and hypermethylation at other specific locus. Our results may be important to unravel the underlying mechanism of tumor multiplicity in the non-hereditary scenario, and provide novel potential biomarkers for identifying high-risk patients and tailoring surveillance strategies.

## Supporting Information

Figure S1 Heatmap showing the 172 most significantly hypermethylated CpG sites that differentiate KRAS mutant (n = 13) versus KRAS wild-type tumors (n = 28) based on the Infinium DNA methylation data. The DNA methylation β-values are represented by using a color scale from red (high DNA methylation) to green (low DNA methylation). Rows represent probes and columns represent tumor samples. Clinical and molecular features (group, gender, tumor location, CIMP-H and *KRAS* mutational status) are represented above the heatmap with horizontal bars.
(TIF)

Table S1 Methylight primers and probes used in this study.
(DOCX)

Table S2 Hypermethylated CpG sites found in multiple versus solitary tumors based on the Infinium DNA methylation assay.
(PDF)

Table S3 Hypermethylated CpG sites found in CIMP-H versus CIMP-0/L tumors based on the Infinium DNA methylation assay.
(PDF)

Table S4 Hypermethylated CpG sites found in *KRAS* mutant versus. *KRAS* wild-type tumors based on the Infinium DNA methylation assay.
(PDF)

Table S5 Functional annotation clustering of differentially methylated genes found in multiple versus solitary tumors based on DAVID analysis.
(PDF)
