# Supplementary figures and images for: Multiple Sporadic Colorectal Cancers Display a Unique Methylation Phenotype
Source: PLoS One. 2014 Mar 18;9(3):e91033. doi: 10.1371/journal.pone.0091033 (PMC3958343; doi:10.1371/journal.pone.0091033)

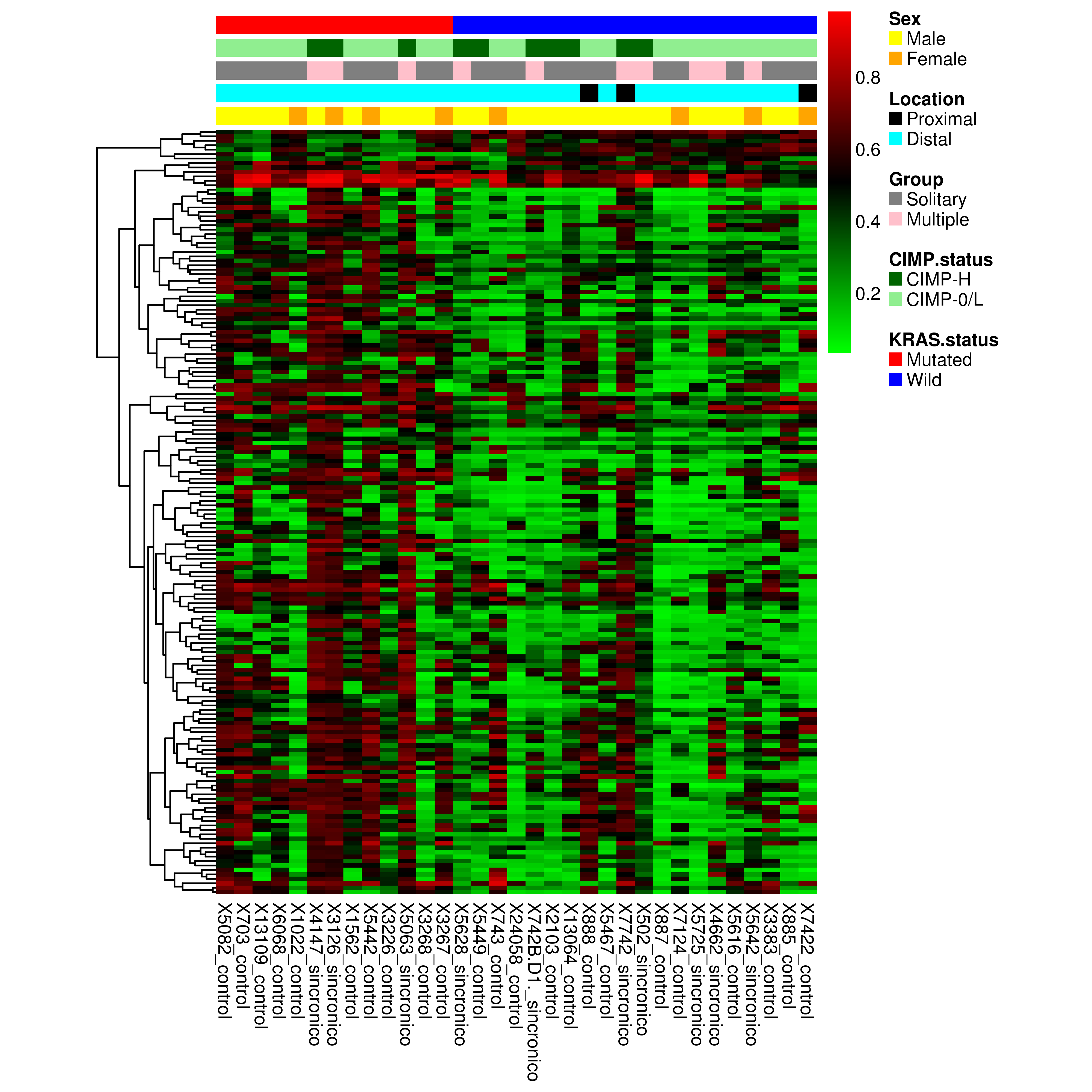

Supplement: Figure S1 — Heatmap showing the 172 most significantly hypermethylated CpG sites that differentiate KRAS mutant (n = 13) versus KRAS wild-type tumors (n = 28) based on the Infinium DNA methylation data. The DNA methylation β-values are represented by using a color scale from red (high DNA methylation) to green (low DNA methylation). Rows represent probes and columns represent tumor samples. Clinical and molecular features (group, gender, tumor location, CIMP-H and KRAS mutational status) are represented above the heatmap with horizontal bars. (TIF) [file pone.0091033.s001.tif]
