# Supplementary material for: Multiple Sporadic Colorectal Cancers Display a Unique Methylation Phenotype
Source: PLoS One. 2014 Mar 18;9(3):e91033. doi: 10.1371/journal.pone.0091033 (PMC3958343; doi:10.1371/journal.pone.0091033)
Supplement: Table S1 — Methylight primers and probes used in this study. (DOCX) [file pone.0091033.s002.docx]

| **Gene** | **Primer sequence** | **Lenght** | **Amplicon size (bp)** |
| --- | --- | --- | --- |
| **MAP1B-F** | **ATAGTAGTCGGTAGGATGGC** | **20** | **95** |
| **MAP1B-R** | **CTAAACGAAATAAACGCCG** | **19** |  |
| **MAP1B-probe** | **6FAM-TATCGAGTCGGAGTCGTTCGG** | **21** |  |
|  |  |  |  |
| **HTRA1-F** | **TGTAGGAATTTTTTTCGGC** | **19** | **79** |
| **HTRA1-R** | **CGCGTCCTTCAAACTAATAA** | **20** |  |
| **HTRA1-probe** | **6FAM-TTACGCGAAGTCGTCGTAGG** | **20** |  |
|  |  |  |  |
| **TIMP3-F** | **GGGTATTCGGAGGGTAGC** | **18** | **86** |
| **TIMP3-R** | **GACGAAACTACTACAACCCGA** | **21** |  |
| **TIMP3-probe** | **6FAM-TTGTTTCGTACGGTTCGGCGG** | **21** |  |
|  |  |  |  |
| **ALOX15-F** | **GGGTTTTTATCGTATTCGC** | **19** | **89** |
| **ALOX15-R** | **CGTACTAACCGACCAACC** | **18** |  |
| **ALOX15-probe** | **6FAM-ATTGGGGTTTCGTTTTATGTCGGTT** | **25** |  |
|  |  |  |  |
| **ALUC4-F** | **GGTTAGGTATAGTGGTTTATATTTGTAATTTTAGTA** | **36** | **98** |
| **ALUC4-R** | **ATTAACTAAACTAATCTTAAACTCCTAACCTCA** | **33** |  |
| **ALUC4-probe** | **6FAM-CCTACCTTAACCTCCC** | **16** |  |

**Supplementary Table 1. MethyLight primers and probes.**
