# Supplementary material for: Multiple Sporadic Colorectal Cancers Display a Unique Methylation Phenotype
Source: PLoS One. 2014 Mar 18;9(3):e91033. doi: 10.1371/journal.pone.0091033 (PMC3958343; doi:10.1371/journal.pone.0091033)
Supplement: Table S2 — Hypermethylated CpG sites found in multiple versus solitary tumors based on the Infinium DNA methylation assay. (PDF) [file pone.0091033.s003.pdf]

Supplementary Table 2. Hypermethylated CpG sites found in multiple versus solitary tumors based on the Infinium DNA methylation assay.

TargetID	Symbol	beta.dif	beta.Multiple	beta.Solitary	p	OR	ci.low	ci.high
cg07380496	MAP1B	0,3637	0,4641	0,1003	0,002654	1254,9369	11,9611	131665,7994
cg02001410	MAP1B	0,333	0,4472	0,1142	0,003897	1606,0865	10,6882	241341,7341
cg04759439	CAST1	0,2256	0,529	0,3034	0,004122	1862,0013	10,8606	319231,2273
cg25920792	HTRA1	0,3035	0,4161	0,1126	0,004872	2398,6273	10,6402	540725,46
cg09872233	ALOX15	0,1928	0,3004	0,1076	0,005856	1114,2539	7,5839	163709,5028
cg22578204	TIMP3	0,2347	0,4226	0,188	0,006627	1474,7133	7,6077	285864,3648
cg10694152	SLC15A1	0,1656	0,3577	0,1921	0,006717	2572,9798	8,7981	752461,4449
cg10193817	IGSF4	0,1587	0,3073	0,1486	0,00676	91531,8551	23,5027	356473720,8
cg16518291	ZNF272	0,1814	0,2831	0,1017	0,007011	28267,5205	16,4309	48631126,76
cg07102705	HTR4	0,2045	0,3741	0,1696	0,007196	3610,1666	9,1876	1418576,238
cg24482234	LASS1	0,1665	0,3745	0,208	0,007336	25091,5196	15,2573	41264414,61
cg02788102	IGSF4	0,1699	0,3182	0,1484	0,008665	897,1135	5,5986	143753,4979
cg25202471	TFAP2A	0,1569	0,3384	0,1815	0,009046	2611,6967	7,1007	960600,1691
cg08797471	DAPK1	0,1784	0,3992	0,2208	0,009347	13019,4907	10,2759	16495647,26
cg08186362	HRH3	0,2058	0,2923	0,0864	0,010079	35878,7767	12,1724	105754539,8
cg20937139	PDGFC	0,206	0,4387	0,2327	0,010549	284,3234	3,7424	21600,8272
cg04434339	ST6GAL2	0,1971	0,3265	0,1294	0,012537	1701,479	4,9491	584962,7842
cg19378133	A2BP1	0,2283	0,4317	0,2034	0,012644	1194,6884	4,5561	313268,3937
cg12893143	UNC5A	0,2047	0,3309	0,1262	0,013685	1045,5234	4,1593	262811,6694
cg19210770	ACCN4	0,2406	0,4125	0,1719	0,013947	296,4668	3,1722	27707,0226
cg16557944	GPX7	0,4424	0,5179	0,0754	0,015266	36,0311	1,9909	652,0963
cg25760229	CACNA1H	0,173	0,2869	0,1139	0,015671	2274,6314	4,3071	1201262,688
cg02879662	HIF3A	0,3146	0,4356	0,121	0,015802	140,4291	2,5323	7787,5294
cg05621401	RET	0,2045	0,3677	0,1632	0,016008	391,3649	3,041	50366,7371
cg15447479	SMO	0,2536	0,3525	0,0989	0,016159	158,0587	2,5533	9784,2791
cg05636175	TNFRSF10C	0,2884	0,5738	0,2855	0,016887	220,7269	2,6367	18477,686
cg20792294	IGF2AS	0,2454	0,6259	0,3805	0,017358	430,0285	2,9101	63546,1738
cg21530890	SOX8	0,2973	0,4117	0,1144	0,017996	118,6858	2,2687	6209,0924
cg18275051	CYB5R1	0,1633	0,3308	0,1675	0,019185	718,6792	2,9234	176678,8069
cg07558455	ANKRD38	0,1739	0,3231	0,1492	0,019441	259,142	2,451	27398,3563
cg24727182	AGXT2L1	0,1559	0,2322	0,0764	0,020384	1815,4514	3,1976	1030743,912
cg05520656	ZNF681	0,227	0,3818	0,1548	0,021474	66,6207	1,8591	2387,3879
cg26195812	DPYSL5	0,2987	0,6082	0,3095	0,022539	75,3057	1,8377	3085,9325
cg23422659	WNT9B	0,1823	0,3442	0,1619	0,023593	319,769	2,1681	47161,555
cg08528984	PRDM16	0,2552	0,3581	0,1029	0,02385	73,7086	1,7685	3072,1517
cg03586879	A2BP1	0,1524	0,27	0,1177	0,024648	2388,7647	2,7	2113378,899
cg13346411	CCKBR	0,3648	0,634	0,2692	0,024983	91,61	1,7642	4757,0376
cg25332298	ELAVL3	0,2615	0,5445	0,283	0,025085	230,857	1,9749	26986,604
cg16428251	SOX14	0,1691	0,4451	0,276	0,025199	397,72	2,1053	75136,5303
cg12439899	TFAP2A	0,2738	0,5104	0,2366	0,02696	104,3653	1,6985	6412,6878
cg15898840	IGFBP3	0,2889	0,6021	0,3132	0,028056	59,1325	1,5517	2253,4497
cg16175263	TNFRSF10C	0,2051	0,6018	0,3967	0,029634	206,3801	1,6935	25150,4217
cg15835825	HTR5A	0,173	0,3108	0,1378	0,02974	572,4671	1,8663	175599,6329
cg27090216	TNFRSF10C	0,3042	0,5198	0,2156	0,030103	61,1433	1,4858	2516,1855
cg11465971	GSH2	0,2839	0,413	0,1291	0,033665	71,2325	1,3904	3649,448
cg17530977	GLI3	0,1891	0,3719	0,1829	0,034984	348,4208	1,5105	80367,9298
cg08853659	CLSTN2	0,4015	0,522	0,1205	0,036409	25,4401	1,2272	527,3786
cg00514895	CPZ	0,1993	0,3499	0,1506	0,037461	113,7457	1,3162	9830,2246
cg25422943	PCDH9	0,4552	0,6055	0,1503	0,038328	41,4925	1,2218	1409,13
cg20256494	CABP7	0,2625	0,4376	0,1751	0,038588	43,3865	1,2188	1544,3959
cg22307908	ZNF264	0,1932	0,3461	0,1529	0,040228	502,8362	1,3195	191624,0021

cg11981631	ABCC8	0,2792	0,4716	0,1924	0,040615	85,2965	1,2092	6016,7773
cg00117172	RUNX3	0,162	0,2954	0,1334	0,041507	112,7527	1,1995	10598,9385
cg00557354	ARHGEF7	0,3049	0,5252	0,2203	0,041822	27,2237	1,1301	655,8285
cg07621046	C10orf82	0,2038	0,6334	0,4296	0,042197	423,6912	1,2376	145045,0716
cg26646370	SHD	0,1572	0,3331	0,1759	0,045192	28,9218	1,0747	778,3061
cg10158080	SOX5	0,2073	0,3434	0,136	0,045537	28,6979	1,0689	770,4776
cg01697732	FAM20A	0,3719	0,6049	0,233	0,04715	16,8359	1,0362	273,5525
cg17054360	MTERF	0,1747	0,2773	0,1026	0,048984	79,3281	1,0197	6171,5191
cg23695504	FLJ45717	0,1331	0,3888	0,2557	0,004779	40920,2327	25,6029	65401286,58
cg01530101	KCNQ1DN	0,1284	0,5557	0,4273	0,005988	1384308,115	57,7851	33162706168
cg25741452	KITLG	0,1062	0,2206	0,1144	0,006511	11995596,84	95,4354	1,50777E+12
cg01718139	UNQ3033	0,1295	0,7418	0,6122	0,006518	1605418169	374,5406	6
cg11993754	ERBB2	0,1108	0,2797	0,1689	0,007192	8580256,629	75,4302	9,76012E+11
cg19646028	C19orf30	0,1116	0,4424	0,3308	0,007992	266995,4457	26,0996	2731323698
cg24662961	IRX3	0,13	0,297	0,167	0,011466	93138,4615	13,0857	662920088,2
cg23095584	GBX2	0,1428	0,3786	0,2358	0,014513	1743,5492	4,3882	692756,6592
cg26055770	PDZRN3	0,1036	0,3016	0,198	0,015003	171983,3286	10,3923	2846183026
cg14223995	UCP1	0,1301	0,2534	0,1233	0,016588	7056,8158	5,0119	9936109,4
cg06626655	RSPO1	0,1371	0,3304	0,1933	0,017314	14490,4849	5,4241	38711119,88
cg19781133	KCNH2	0,1283	0,2446	0,1163	0,017857	1479,5759	3,5229	621411,5289
cg24120841	THRB	0,1026	0,2187	0,1161	0,018041	1369192894	36,6443	5
cg15843823	ALOX15	0,1268	0,3291	0,2022	0,018152	430,1331	2,8102	65836,5456
cg18815943	FOXE3	0,1065	0,2152	0,1088	0,019373	664,5369	2,862	154303,011
cg08700306	LRP3	0,1006	0,4214	0,3208	0,021399	882263,7035	7,6021	1,02392E+11
cg15774153	FGF19	0,1054	0,2191	0,1137	0,021958	1919,077	2,9819	1235057,472
cg24396745	HCN4	0,1421	0,5402	0,398	0,022103	293,3574	2,261	38061,9778
cg24680602	ZNF232	0,147	0,3003	0,1533	0,023171	357,5413	2,2352	57191,4526
cg00121640	ASTN2	0,1289	0,3087	0,1798	0,023713	746,933	2,4176	230770,944
cg12373771	CECR6	0,1229	0,3228	0,1999	0,024764	2032,9931	2,6308	1571020,873
cg13344740	DLX5	0,1381	0,5722	0,4341	0,026562	1565,0557	2,3523	1041260,557
cg03462055	TUBB2B	0,1404	0,2358	0,0954	0,027289	524627,4102	4,3759	62897756279
cg07098866	MEGF11	0,1014	0,2016	0,1002	0,028033	1073,9644	2,1223	543468,5135
cg08914623	ALX4	0,1361	0,2271	0,091	0,029001	190,2514	1,7113	21151,3821
cg18403396	B3GAT2	0,133	0,2777	0,1448	0,030826	498,3306	1,7743	139959,7986
cg17183991	HS6ST3	0,1397	0,2423	0,1026	0,031181	79,9756	1,486	4304,1748
cg19237753	PTPNS1	0,1162	0,2239	0,1077	0,031246	6166,0066	2,1943	17326600,6
cg13384396	ADCY5	0,141	0,3648	0,2238	0,032826	79,0665	1,4288	4375,4635
cg18618334	CXCL12	0,1038	0,3176	0,2139	0,034958	1454,5096	1,6721	1265200,064
cg15057581	PTPNS1	0,1261	0,2565	0,1303	0,035027	163,4288	1,4305	18671,2775
cg05674944	SLC30A2	0,1169	0,3145	0,1976	0,035345	392,5616	1,5066	102283,2206
cg02441647	COL8A1	0,1201	0,351	0,2308	0,035687	602,6012	1,5345	236640,4879
cg15309006	LOC63928	0,1319	0,2662	0,1342	0,036033	377,4988	1,4719	96815,0896
cg12594641	MGC52057	0,1218	0,2737	0,1519	0,036862	1050,3588	1,5287	721717,5635
cg14603345	BTBD3	0,1156	0,4487	0,3331	0,037104	1013,9105	1,5127	679595,0722
cg16933388	BSN	0,1349	0,2968	0,162	0,038942	299,3196	1,3357	67073,2788
cg12949975	GDF7	0,1018	0,2097	0,1079	0,040116	91,7061	1,2261	6859,1841
cg26465611	MEGF10	0,1227	0,495	0,3723	0,04072	151,4072	1,2361	18545,6073
cg05942574	CACNA1G	0,1096	0,3516	0,2419	0,042166	708,0897	1,2615	397469,5667
cg18952560	PTPNS1	0,121	0,2427	0,1217	0,042205	79,9011	1,1668	5471,5839
cg07408456	PGLYRP2	0,1036	0,4866	0,383	0,043365	15260,7275	1,3326	174762068
cg17162024	UNQ9433	0,1308	0,6703	0,5395	0,04906	32,4275	1,0144	1036,5856
