# Supplementary material for: Multiple Sporadic Colorectal Cancers Display a Unique Methylation Phenotype
Source: PLoS One. 2014 Mar 18;9(3):e91033. doi: 10.1371/journal.pone.0091033 (PMC3958343; doi:10.1371/journal.pone.0091033)
Supplement: Table S3 — Hypermethylated CpG sites found in CIMP-H versus CIMP-0/L tumors based on the Infinium DNA methylation assay. (PDF) [file pone.0091033.s004.pdf]

Supplementary Table 3. Hypermethylated CpG sites found in CIMP-H versus CIMP-0/L tumors based on the Infinium DNA methylation assay.

TargetID	Symbol	beta.dif	beta.CIMP-H	beta.CIMP-L/0	p	OR	ci.low	ci.high
cg13384396	ADCY5	0,2941	0,5064	0,2123	0,001081	40194,2172	69,7236	23171145,88
cg09156233	BMPR1B	0,2793	0,4363	0,157	0,001104	1936845,305	323,725	11588135569
cg15309006	LOC63928	0,1783	0,3061	0,1278	0,001239	290685652,6	2126,0627	3,9744E+13
cg11896271	PANX2	0,2814	0,4663	0,1849	0,001311	17863,8323	45,5662	7003357,54
cg07558455	ANKRD38	0,2344	0,3706	0,1362	0,001364	1218142,537	229,5061	6465497975
cg14580567	HHIP	0,377	0,5022	0,1253	0,001487	1707,1756	17,3136	168332,4392
cg04759439	CAST1	0,2736	0,5773	0,3037	0,001734	3978,9664	22,251	711526,0922
cg02250594	ONECUT2	0,2602	0,5847	0,3245	0,001893	19318,2687	38,188	9772589,523
cg18454685	CACNA1G	0,3456	0,5077	0,1621	0,001984	2928,9508	18,6079	461026,4384
cg25920792	HTRA1	0,3495	0,4613	0,1118	0,002003	73115,0455	60,0698	88993315,36
cg20937139	PDGFC	0,2459	0,4643	0,2184	0,002108	10905,2101	29,0934	4087652,47
cg22578204	TIMP3	0,3028	0,4754	0,1726	0,002495	177340,5451	70,2741	447528474
cg07102705	HTR4	0,3078	0,4726	0,1648	0,002735	364012,5701	83,7695	1581782852
cg23695504	FLJ45717	0,1972	0,4537	0,2565	0,00285	1420502,272	129,0382	15637433080
cg01530101	KCNQ1DN	0,1716	0,5991	0,4275	0,002988	37597532,18	375,3724	3,76579E+12
cg24120841	THRB	0,1155	0,2269	0,1114	0,003239	4	811175,372	2
cg25202471	TFAP2A	0,2093	0,3903	0,181	0,003264	91024,747	45,194	183331805,3
cg23771929	FREQ	0,1063	0,3548	0,2485	0,003302	2658391623	1373,0899	5
cg23146358	CDKN1C	0,114	0,1821	0,0682	0,003454	25579575201	2702,424	2
cg01468621	BRSK2	0,2907	0,5211	0,2304	0,003509	813,3441	9,0437	73148,3531
cg26055770	PDZRN3	0,123	0,3249	0,2018	0,00364	28162994283	2547,5787	3
cg14717170	SLITRK3	0,1062	0,2909	0,1847	0,00375	691490959,8	728,0856	6,56736E+14
cg00666746	SYDE1	0,1376	0,3765	0,2388	0,003755	12525658,67	198,4158	7,90724E+11
cg18618334	CXCL12	0,1922	0,3972	0,2049	0,003762	1394250,107	97,2866	19981509248
cg20380069	MSI1	0,1461	0,293	0,1469	0,00394	1,327E+13	15895,7782	1
cg22747092	BTBD14A	0,1026	0,3525	0,2499	0,004011	13448628648	1695,1805	1
cg22375192	IGF1R	0,1998	0,3276	0,1278	0,004085	383335,4111	59,2247	2481159791
cg01697732	FAM20A	0,4785	0,6321	0,1536	0,004116	115,4331	4,5032	2958,9345
cg25957124	DNAH3	0,1089	0,3249	0,216	0,004152	1283530220	759,1609	2
cg03310469	SIX2	0,1601	0,2347	0,0746	0,004167	20802950045	1820,0809	2
cg02441647	COL8A1	0,1839	0,4081	0,2242	0,004229	17157,1371	21,5354	13668971,79
cg08528984	PRDM16	0,3063	0,4271	0,1208	0,004493	685,6884	7,5809	62020,0378
cg12277666	TDRD5	0,1119	0,33	0,2181	0,004513	16697649,34	172,9228	1,61235E+12
cg12949975	GDF7	0,2469	0,3424	0,0954	0,004759	2100,3445	10,3652	425600,4664
cg00468146	ID4	0,1327	0,3589	0,2262	0,004772	109245,7372	34,6021	344910169,8
cg25741452	KITLG	0,1116	0,2273	0,1157	0,004773	721655623,4	508,013	1
cg25088874	BMPR1B	0,1207	0,246	0,1253	0,004848	4,52238E+11	3514,976	5
cg23166362	PIK3CD	0,4237	0,554	0,1303	0,00487	249,9191	5,3532	11667,6693
cg06790324	GRB10	0,118	0,2105	0,0924	0,004922	265511817,1	356,98	1,9748E+14
cg05595345	ARRDC4	0,1028	0,2533	0,1505	0,004958	1459690758	591,1031	3
cg04434339	ST6GAL2	0,2684	0,3998	0,1314	0,004995	3501327,278	94,492	1,29739E+11
cg12594641	MGC52057	0,2017	0,3668	0,1651	0,005003	55569,9207	27,0155	114305194,3
cg25766046	ROR2	0,2453	0,4443	0,199	0,005031	671,488	7,1074	63440,2087
cg20339650	IGF2	0,1422	0,3976	0,2553	0,005096	8840147,051	121,7898	6,41665E+11
cg23748737	SCARF2	0,2522	0,4021	0,15	0,005113	8428,3492	15,0514	4719620,694
cg15439862	DSC3	0,1416	0,3667	0,2251	0,00513	55784467,49	209,7298	1,48377E+13
cg20616414	WNK2	0,1673	0,3792	0,2119	0,005134	4816790,084	100,5589	2,30725E+11
cg11993754	ERBB2	0,1152	0,2842	0,1689	0,005142	72964299,58	226,4881	2,35058E+13
cg02090283	CNTNAP3	0,2382	0,3262	0,088	0,005217	625,6947	6,8277	57338,8319
cg24396745	HCN4	0,229	0,5953	0,3663	0,005246	13801,5906	17,1101	11132832,15
cg11832722	DSC3	0,1056	0,3366	0,231	0,005276	1	34382,7026	9
cg15774153	FGF19	0,1647	0,2769	0,1122	0,005296	3	9496731,694	9

cg05674944	SLC30A2	0,1568	0,3539	0,197	0,005333	256775,3822	40,1958	1640312394
cg17183991	HS6ST3	0,1747	0,2518	0,0771	0,005345	450,2898	6,1151	33157,5488
cg21380842	KCNJ6	0,2192	0,3137	0,0945	0,005364	11081,3215	15,7577	7792739,162
cg23090046	KNS2	0,1	0,3889	0,2889	0,00542	1,6695E+12	4058,1082	6
cg02166532	IGF2	0,1286	0,5182	0,3896	0,005446	1	62254,127	5
cg19177941	ALDH1A3	0,1337	0,4505	0,3167	0,005474	7,04011E+14	23504,5407	2
cg02111587	TACR1	0,153	0,2953	0,1423	0,005521	9926,1821	14,9213	6603265,989
cg18815943	FOXE3	0,1542	0,2645	0,1103	0,005532	8025,4314	13,9982	4601126,88
cg10694152	SLC15A1	0,2252	0,4189	0,1937	0,005665	6424840,622	96,5773	4,27415E+11
cg24482234	LASS1	0,1681	0,3704	0,2023	0,005669	42691,8847	22,3731	81463868,53
cg08797471	DAPK1	0,1657	0,3866	0,2209	0,005808	36863,5	20,9842	64759094,47
cg12893143	UNC5A	0,299	0,4238	0,1248	0,005991	10411540,66	103,0216	1,05221E+12
cg24662961	IRX3	0,1375	0,314	0,1766	0,00602	446269241,5	300,219	6,6337E+14
cg07845566	B3GAT1	0,3306	0,4065	0,076	0,006056	403,5998	5,5603	29295,8344
cg22919728	CHST13	0,1113	0,3875	0,2762	0,006087	1468686,102	57,6216	37434558867
cg10000775	PDLIM3	0,2266	0,4173	0,1907	0,00615	701,3296	6,4558	76189,5828
cg04532952	CA4	0,1346	0,3101	0,1755	0,00625	9389,3968	13,339	6609264,152
cg25027167	DGAT1	0,1384	0,3803	0,2419	0,00626	39805,4368	20,0515	79020206,37
cg25234611	VWA1	0,1331	0,2357	0,1026	0,006279	6628499,92	84,9437	5,17249E+11
cg22377389	GJB6	0,2032	0,412	0,2088	0,006483	10458,6547	13,3529	8191735,589
cg09952204	RASGRF2	0,1118	0,1894	0,0775	0,006567	1110036979	333,2496	3
cg04684516	SNCAIP	0,1218	0,244	0,1222	0,006574	63349,4971	21,8178	183939523,6
cg16632715	HOXD11	0,1125	0,3396	0,2272	0,00668	2295822,668	58,153	90636744234
cg15105703	NIP	0,1338	0,2789	0,1451	0,006697	7390842909	543,818	1
cg23922708	PARD6G	0,1353	0,2269	0,0916	0,006749	403619517,2	239,5912	6,79944E+14
cg14223995	UCP1	0,1276	0,2506	0,1229	0,006781	66060,0763	21,4136	203792582,6
cg21965997	DRD1IP	0,243	0,345	0,1021	0,006834	10000,5855	12,6344	7915808,461
cg21481775	NKX3-1	0,1524	0,2929	0,1405	0,006956	13924,8253	13,6335	14222395,99
cg02515725	PDLIM3	0,1448	0,3161	0,1713	0,007053	1942,8724	7,8756	479297,1474
cg22368262	C10orf35	0,1529	0,302	0,1491	0,007056	315253,6536	31,5148	3153596225
cg27049761	B3GNT4	0,3723	0,4667	0,0945	0,007072	244,6864	4,4716	13389,2339
cg12515638	SFRP4	0,2355	0,3425	0,107	0,007199	858,42	6,2256	118364,2191
cg27626299	EVX1	0,101	0,2301	0,1291	0,007316	1	76805,5099	2
cg08853659	CLSTN2	0,4912	0,5981	0,107	0,007329	88,7052	3,3431	2353,6779
cg21321735	KIF1A	0,2765	0,3577	0,0811	0,007336	260,0461	4,4631	15151,8213
cg12373771	CECR6	0,2128	0,4063	0,1936	0,00741	182804,5737	25,7414	1298198603
cg08186362	HRH3	0,1994	0,2854	0,086	0,007476	257598,4988	27,9347	2375434662
cg24417499	HPCA	0,1075	0,3236	0,2161	0,007505	164038,4519	24,6547	1091418654
cg24727182	AGXT2L1	0,1627	0,2363	0,0736	0,007515	5177,3257	9,7921	2737371,905
cg16557944	GPX7	0,3683	0,4371	0,0688	0,007625	68,9498	3,0764	1545,3526
cg15887846	CST6	0,1011	0,4455	0,3445	0,007689	347167,8878	29,2716	4117485661
cg08914623	ALX4	0,2553	0,3422	0,0868	0,007799	900,5793	5,9991	135194,9467
cg05621401	RET	0,2602	0,4212	0,1611	0,007903	1163,9914	6,3625	212946,6229
cg07452799	PARD3	0,1022	0,1893	0,0871	0,007942	92321497,79	121,3827	7,02181E+13
cg06263495	ASCL2	0,1047	0,2757	0,171	0,0081	3851289,298	51,3987	2,88576E+11
cg00775197	MGC33600	0,1209	0,2689	0,148	0,008178	257030,1514	25,1545	2626350547
cg24680602	ZNF232	0,2012	0,3539	0,1527	0,008292	1611,0507	6,6998	387397,1322
cg05260966	TIMP3	0,1476	0,4069	0,2593	0,008409	75930,1128	17,804	323824249
cg15898840	IGFBP3	0,3529	0,6651	0,3122	0,008511	1196,9882	6,0976	234973,132
cg00290506	CNIH3	0,318	0,4609	0,1429	0,008541	185,1547	3,7813	9066,3685
cg00351011	LASS4	0,2807	0,3811	0,1004	0,008576	206,7083	3,8805	11011,0125
cg22750254	CCDC3	0,2157	0,3529	0,1373	0,008586	5877,0929	9,0828	3802802,387
cg12220493	TITF1	0,1025	0,2214	0,119	0,008595	276320,3614	24,1443	3162364088
cg22571530	NFASC	0,1335	0,2765	0,143	0,008632	56182,9734	16,0351	196850846,8
cg15057581	PTPNS1	0,2029	0,3259	0,123	0,008731	1342,6576	6,1684	292253,075
cg05270634	RND2	0,2515	0,5015	0,2501	0,008736	2217,4785	6,9986	702595,1794
cg22772878	DIRAS1	0,1296	0,4836	0,354	0,00877	20638,6794	12,2477	34778285,99
cg22396755	RAP1GA1	0,1146	0,2651	0,1505	0,008936	22748,6065	12,3245	41989612,31
cg12770741	NXN	0,1242	0,6486	0,5243	0,008963	11302,2812	10,3154	12383544,2
cg01352108	KCNK4	0,1197	0,4963	0,3766	0,009077	129584,2056	18,7091	897534233,4

cg07380496	MAP1B	0,3075	0,4054	0,0979	0,009094	120,8457	3,2938	4433,7555
cg07109287	LHX2	0,2578	0,4826	0,2248	0,009188	201,4097	3,7196	10905,8322
cg23397015	INHBB	0,1106	0,3177	0,207	0,009272	704047,13	27,7095	17888504830
cg02409351	CART1	0,2651	0,395	0,1299	0,009355	485,9561	4,5754	51613,6908
cg15447479	SMO	0,333	0,4297	0,0966	0,009369	201,9643	3,6842	11071,3927
cg24881834	ME1	0,1024	0,3295	0,2271	0,009529	4051198,827	40,937	4,00914E+11
cg26195812	DPYSL5	0,3813	0,6418	0,2605	0,009531	201,6106	3,6491	11138,7593
cg00234616	TLX2	0,1257	0,3297	0,2041	0,009558	711587,25	26,6691	18986659698
cg03048535	FOXN4	0,1337	0,2118	0,0781	0,009623	8898653,521	48,819	1,62203E+12
cg26363196	ST6GALNAC3	0,1256	0,2602	0,1346	0,009676	8925,0744	9,0737	8778924,405
cg10193817	IGSF4	0,1441	0,3031	0,1589	0,009734	13944719,01	53,426	3,63971E+12
cg00625653	WNT7A	0,368	0,5527	0,1847	0,009751	108,8233	3,1059	3812,9628
cg11115702	SPIN1	0,1317	0,2651	0,1334	0,009772	21404515,52	58,8657	7,78303E+12
cg19781133	KCNH2	0,1328	0,25	0,1172	0,010002	12356,8147	9,5119	16052634,95
cg08315770	KCNK17	0,2213	0,3908	0,1695	0,010007	195,2942	3,528	10810,6536
cg06637774	P2RY6	0,1167	0,4292	0,3125	0,010038	108777,4141	15,932	742689506,1
cg23422659	WNT9B	0,1564	0,3084	0,152	0,010269	1885,3133	5,9457	597815,5879
cg04796162	IGFBP3	0,2612	0,4573	0,1961	0,010377	163,8095	3,3191	8084,663
cg01805540	CACNB2	0,1145	0,2251	0,1107	0,010481	70487,0258	13,6678	363511813,6
cg01899253	FLT1	0,1109	0,3985	0,2877	0,010735	98165,9878	14,3506	671507055,6
cg21303386	RGS7	0,1528	0,2931	0,1402	0,010915	4951,5236	7,0761	3464835,454
cg16352283	FAM46B	0,1925	0,5464	0,354	0,011082	1850,9452	5,5751	614513,5432
cg21530890	SOX8	0,3316	0,4424	0,1109	0,011123	200,6538	3,3493	12020,9541
cg14706178	FLJ32786	0,1563	0,219	0,0627	0,011199	1599,6349	5,3479	478475,4938
cg09254939	KLK10	0,139	0,2643	0,1253	0,011428	25690,8815	9,8326	67125614,95
cg08261094	SFRP4	0,2561	0,4963	0,2401	0,011572	193,9454	3,25	11573,6225
cg10644361	MIPOL1	0,1229	0,2716	0,1486	0,011594	5439,8699	6,8406	4325939,08
cg18275051	CYB5R1	0,1557	0,3167	0,161	0,011629	1664,268	5,2368	528911,4504
cg20256494	CABP7	0,3103	0,4829	0,1726	0,011647	135,8982	2,9911	6174,422
cg14662379	KIF1A	0,3508	0,5768	0,226	0,011737	255,1346	3,4264	18997,7926
cg24317255	RGS17	0,1511	0,2483	0,0972	0,011882	1577,1014	5,0858	489061,8901
cg04686412	PSD2	0,1063	0,3045	0,1982	0,012028	5350,4598	6,5851	4347284,457
cg23854009	ZNF530	0,1534	0,2856	0,1322	0,012217	3318,6974	5,8478	1883407,842
cg21296230	GREM1	0,1134	0,3269	0,2135	0,012241	3688,7036	5,9733	2277875,072
cg17252960	ID4	0,1288	0,3439	0,215	0,012297	681,7844	4,123	112741,5944
cg16731240	ZNF577	0,3287	0,6465	0,3178	0,012549	167,124	3,0036	9299,0065
cg14603345	BTBD3	0,1213	0,4609	0,3396	0,012579	16797,2579	8,0668	34976227,88
cg17740399	IPF1	0,11	0,2047	0,0947	0,012617	7662150,872	29,8558	1,9664E+12
cg02001410	MAP1B	0,233	0,3415	0,1086	0,012897	158,0913	2,9221	8552,9637
cg03251079	HMGA2	0,1065	0,2433	0,1367	0,01298	866271,6049	17,9164	41884978155
cg02016419	TEKT3	0,1161	0,4703	0,3542	0,013058	156789,9409	12,3884	1984356162
cg04993257	PLAC2	0,117	0,4983	0,3813	0,01327	36325,5635	8,9343	147694149,1
cg18342279	ZAR1	0,1069	0,1923	0,0854	0,013288	173126,6632	12,3507	2426818132
cg15843823	ALOX15	0,1216	0,3245	0,2029	0,013327	1137,9396	4,3238	299485,4416
cg11648289	EN2	0,1158	0,2092	0,0934	0,013378	6775,3038	6,2432	7352739,796
cg02503850	ADAMTS14	0,3401	0,6196	0,2795	0,01346	431,6108	3,5098	53077,2106
cg21309147	STAC2	0,446	0,5766	0,1306	0,013537	88,0866	2,5187	3080,6251
cg21905630	GSH2	0,1081	0,2549	0,1468	0,01366	20562,265	7,6769	55075184,98
cg12420104	DMRT3	0,2365	0,4542	0,2177	0,013734	76,8796	2,4314	2430,92
cg18501026	DOCK3	0,1456	0,4697	0,324	0,013995	345,5298	3,2648	36569,6466
cg20239740	SYT5	0,2527	0,3837	0,131	0,014006	375,0778	3,3176	42404,9115
cg02788102	IGSF4	0,2328	0,3825	0,1497	0,014054	2553,8466	4,8753	1337785,92
cg19224837	GSC	0,1579	0,254	0,0961	0,014351	1699,7962	4,4095	655238,672
cg14046986	RCSD1	0,1204	0,1944	0,074	0,014658	273,0597	3,0191	24697,045
cg12388309	PAK7	0,2689	0,5986	0,3297	0,014864	152,013	2,6677	8662,0939
cg18952560	PTPNS1	0,1747	0,2961	0,1214	0,014882	339,01	3,1176	36864,3711
cg10978355	CKMT2	0,1111	0,3882	0,2771	0,014918	480,8893	3,3318	69409,1066
cg26780404	COL12A1	0,1019	0,1984	0,0966	0,015076	925302,7174	14,2931	59901820285
cg21351994	EMX1	0,1254	0,1978	0,0723	0,015252	9639,6781	5,8317	15934091,88
cg02672220	SIM2	0,1006	0,2952	0,1947	0,015259	3024302844	66,3351	1

cg18959478	PRKCDBP	0,1164	0,2311	0,1147	0,015408	2	11926,3426	4
cg23472215	GSTM3	0,3362	0,4584	0,1222	0,015636	55,2835	2,1364	1430,5588
cg18489434	VASH1	0,1702	0,2293	0,0591	0,015707	1045,4704	3,7116	294488,1424
cg08185241	G0S2	0,1558	0,3819	0,2261	0,015815	408,7761	3,0934	54018,0784
cg04528819	KLF14	0,111	0,286	0,175	0,015995	1203,0268	3,7514	385796,116
cg18403396	B3GAT2	0,1662	0,298	0,1318	0,016131	1447,4314	3,8536	543657,5743
cg12998491	FAM78A	0,4509	0,5064	0,0554	0,016589	860863,1756	12,0073	61719373513
cg02244695	HCA112	0,1006	0,2407	0,1401	0,016662	15068699,43	20,0263	1,13384E+13
cg08211091	FLJ37478	0,1632	0,4194	0,2562	0,016687	255,5706	2,7296	23928,8031
cg26646370	SHD	0,2561	0,4303	0,1742	0,017036	68,265	2,1255	2192,4779
cg03663215	ADCY2	0,1059	0,2366	0,1307	0,017146	10811,9261	5,2106	22434650,28
cg12439899	TFAP2A	0,2736	0,4868	0,2131	0,017412	166,2656	2,4565	11253,317
cg13344740	DLX5	0,1433	0,5752	0,4319	0,017678	1986,3822	3,7431	1054130,151
cg06552037	INHA	0,3988	0,5193	0,1205	0,018471	76,6737	2,0741	2834,4375
cg02536286	TFAP2C	0,4686	0,5866	0,118	0,018525	23,7844	1,7015	332,4604
cg14116122	CART1	0,1873	0,2812	0,0939	0,018578	359,321	2,6771	48228,6062
cg02154186	PNMA2	0,1836	0,4315	0,248	0,018839	1850,4131	3,4736	985732,8389
cg21918500	ZNF124	0,1928	0,3736	0,1809	0,019013	2095,2522	3,5128	1249738,148
cg13694867	SIM2	0,1109	0,2087	0,0977	0,019164	4301,6622	3,9188	4721870,394
cg13230197	CSPG4	0,148	0,4189	0,2709	0,019327	14663,3613	4,7358	45402189,91
cg19283196	SLC10A4	0,1604	0,3442	0,1839	0,019423	585,464	2,7977	122518,8275
cg07850604	INSM2	0,1113	0,2099	0,0986	0,019979	1916,3099	3,2918	1115565,777
cg22231902	EN1	0,3476	0,5194	0,1718	0,020268	69,8624	1,9369	2519,8666
cg24719601	PHOX2B	0,1246	0,3337	0,2091	0,020884	27132,5041	4,6983	156690805,1
cg03160135	VGLL2	0,1399	0,3572	0,2173	0,021198	453,4422	2,4952	82401,4848
cg25242557	PAX6	0,1757	0,4691	0,2935	0,021361	1628,5432	2,9969	884971,4713
cg15536490	TFAP2C	0,2222	0,389	0,1668	0,021602	102,1266	1,9725	5287,5977
cg04451770	ENTPD1	0,1184	0,4382	0,3198	0,021631	24206,2067	4,3945	133336023,1
cg17788682	ARNT2	0,1432	0,2235	0,0803	0,021774	919165,9892	7,396	1,14233E+11
cg01557297	SLC22A17	0,1973	0,2705	0,0732	0,021795	92,3998	1,9327	4417,4518
cg19378133	A2BP1	0,2415	0,4404	0,1989	0,021957	731,3547	2,594	206196,519
cg00024396	ELOVL5	0,1799	0,3249	0,1451	0,0226	555,8618	2,429	127203,1331
cg26372517	TFAP2E	0,1256	0,4354	0,3098	0,022882	5016,2862	3,2582	7723045,69
cg02927346	RASL10B	0,1798	0,4947	0,3149	0,023318	971,6899	2,5467	370750,8109
cg08820801	FBXO17	0,1247	0,6638	0,5391	0,023427	1369,4552	2,6551	706330,9685
cg19995014	APLP1	0,2023	0,3238	0,1215	0,023446	903,1112	2,5077	325238,7194
cg20881054	VASH1	0,1255	0,2043	0,0788	0,023517	380,9121	2,2259	65184,2317
cg17907567	HAMP	0,1229	0,4285	0,3057	0,023636	2788,5151	2,8931	2687755,44
cg09874752	SFRP5	0,414	0,5144	0,1004	0,023699	44,7502	1,6612	1205,528
cg03586879	A2BP1	0,1763	0,2912	0,1149	0,023868	3837,9935	2,9839	4936625,127
cg15242570	CTSL	0,1352	0,2888	0,1536	0,023977	300,5888	2,1213	42594,2158
cg15700739	HOXC5	0,1069	0,36	0,2531	0,024081	496,9921	2,2576	109410,6587
cg14920846	NAV1	0,1097	0,5758	0,4662	0,024159	34059,7236	3,9108	296631475,2
cg11530960	DMRT2	0,2634	0,4073	0,144	0,024346	139,1797	1,8953	10220,6994
cg14155416	L3MBTL4	0,25	0,4152	0,1652	0,024501	111,3077	1,833	6759,2368
cg07098866	MEGF11	0,1766	0,2704	0,0939	0,024586	9383,6397	3,2267	27289133,87
cg19210770	ACCN4	0,2249	0,3908	0,166	0,025096	89,7327	1,7543	4589,963
cg16539629	C14orf132	0,1979	0,3398	0,1419	0,025831	87,2297	1,7141	4439,0143
cg18793806	ZNF514	0,1586	0,4871	0,3285	0,025982	5676,2941	2,8143	11448712,75
cg10247252	GALR2	0,1892	0,3963	0,2071	0,026071	199,831	1,8801	21239,0918
cg14795968	ACADL	0,1652	0,3349	0,1697	0,026132	259,6794	1,9359	34833,3395
cg00121640	ASTN2	0,1434	0,3217	0,1782	0,026139	634,5492	2,1522	187092,8571
cg01920829	CDK5R2	0,2051	0,5254	0,3202	0,026836	234,8878	1,8704	29497,8921
cg19332710	RIMS4	0,4865	0,6112	0,1246	0,026852	32,9133	1,4925	725,8373
cg18678121	SEC61A2	0,1393	0,8164	0,6772	0,026894	1050,9537	2,2159	498446,643
cg19237753	PTPNS1	0,1217	0,2292	0,1075	0,0273	1665,5531	2,2954	1208517,551
cg00565688	TP73	0,2746	0,5531	0,2784	0,027522	126,3961	1,709	9348,2021
cg03682712	LOXL1	0,1105	0,3628	0,2523	0,027579	6214,75	2,6232	14723827,85
cg13643796	ADAMTS3	0,1386	0,2598	0,1212	0,027704	162,3921	1,7478	15088,146
cg07766612	SLC30A2	0,2706	0,4209	0,1503	0,027743	114,587	1,6804	7813,5581

cg12078929	SERHL	0,1169	0,2453	0,1284	0,027788	375,9226	1,9109	73954,119
cg01322134	WNT3A	0,329	0,5869	0,2579	0,027799	48,4481	1,5274	1536,7426
cg12739034	PTGER3	0,1092	0,2121	0,1029	0,02781	194,0243	1,7766	21190,1928
cg22469841	FSTL1	0,1735	0,2446	0,0712	0,027848	142,6213	1,7161	11853,2281
cg15640375	PRG2	0,2956	0,594	0,2984	0,028242	99,7048	1,6335	6085,6439
cg12507125	WWTR1	0,1513	0,2709	0,1196	0,028736	70,9284	1,5567	3231,7414
cg04478795	SMO	0,1188	0,3118	0,1931	0,028953	730,8666	1,9675	271493,6874
cg04969808	WNT7A	0,2963	0,4591	0,1629	0,029038	58,3377	1,515	2246,4157
cg11897314	LOC51334	0,333	0,4966	0,1637	0,029148	42,673	1,464	1243,8585
cg09276363	KCND3	0,1406	0,5361	0,3956	0,029769	1405,3601	2,0361	970023,9988
cg02440177	ZNF702	0,2386	0,3881	0,1496	0,030162	51,4326	1,4594	1812,6064
cg05636175	TNFRSF10C	0,3153	0,5962	0,281	0,03031	67,0222	1,4918	3011,0344
cg22757447	IMPACT	0,1185	0,324	0,2055	0,030451	165,9741	1,6199	17005,7262
cg25738273	DUSP26	0,3557	0,4659	0,1102	0,03053	55,9812	1,4595	2147,282
cg07935568	MLNR	0,3044	0,5306	0,2262	0,03057	63,4418	1,4754	2728,0436
cg25760229	CACNA1H	0,1732	0,2976	0,1244	0,030692	374,8167	1,7357	80938,687
cg00892798	NGFR	0,2863	0,4071	0,1208	0,030778	47,494	1,4296	1577,8158
cg04970352	ALX4	0,2274	0,3603	0,1329	0,031783	51,3014	1,4095	1867,1633
cg00576250	ZNF663	0,1528	0,3039	0,1511	0,032456	1310,4814	1,8222	942470,1631
cg03664800	LOXL2	0,254	0,3791	0,1251	0,032716	61,7042	1,4035	2712,8858
cg09660171	LMX1B	0,1949	0,3784	0,1835	0,032824	196,2532	1,5389	25027,7488
cg11612345	SMOC2	0,1025	0,3343	0,2318	0,032965	385,6243	1,619	91849,5438
cg09595479	PRPH	0,1379	0,4398	0,3019	0,033218	77,395	1,4136	4237,4576
cg19616230	SLC34A2	0,1959	0,3227	0,1268	0,033254	51,9837	1,3685	1974,7044
cg03464655	FLJ39822	0,129	0,4387	0,3097	0,033594	675,2424	1,6582	274971,3711
cg02498063	CCDC60	0,1298	0,5323	0,4025	0,034428	3729,7109	1,8275	7611877,634
cg17226343	DIRAS1	0,1015	0,2716	0,1702	0,035805	2969,8738	1,6992	5190800,217
cg09542745	SFRP5	0,1152	0,2518	0,1367	0,036178	81,4762	1,3277	4999,7946
cg09009111	EMILIN2	0,3728	0,474	0,1012	0,036389	29,4861	1,2391	701,6431
cg14312526	FOXL2	0,2858	0,4673	0,1815	0,036412	61,4415	1,2975	2909,409
cg09721427	HHEX	0,1443	0,387	0,2426	0,036521	220,6466	1,4026	34709,2806
cg14913925	DKFZp727G131	0,1813	0,3752	0,194	0,036645	164,6286	1,3728	19742,6246
cg16786703	ADAM8	0,2185	0,3126	0,0941	0,03665	66,4166	1,2974	3399,9295
cg01718139	UNQ3033	0,1291	0,7512	0,622	0,037058	5539,9212	1,6777	18293792,8
cg17558126	RASSF5	0,1216	0,3534	0,2319	0,037361	185,5621	1,3576	25364,2132
cg00557354	ARHGEF7	0,4404	0,6452	0,2048	0,037513	27,656	1,2114	631,3715
cg13916742	SCGB1D1	0,1034	0,7591	0,6557	0,038145	3495,0937	1,562	7820510,658
cg25549459	POU3F3	0,158	0,3061	0,1481	0,038249	267,2248	1,3534	52763,7495
cg20895028	CDH26	0,1376	0,703	0,5654	0,038352	849,5783	1,436	502647,0302
cg11319389	C20orf100	0,1053	0,451	0,3457	0,038426	278,1114	1,3497	57305,1356
cg16175263	TNFRSF10C	0,2566	0,6463	0,3898	0,038813	102,1148	1,2684	8220,783
cg09306675	EYA2	0,1949	0,4437	0,2488	0,039319	84,4954	1,2425	5745,9279
cg14883392	ASCL1	0,254	0,4168	0,1628	0,039824	245,6332	1,2917	46709,065
cg22309489	OKL38	0,1359	0,8281	0,6923	0,040122	1117,5059	1,3722	910079,8565
cg09945801	WRN	0,1544	0,6584	0,5039	0,04114	303,0788	1,2584	72993,7368
cg18123948	GATA4	0,106	0,2738	0,1678	0,041681	85,1233	1,1822	6129,0096
cg09949775	COMP	0,3086	0,6216	0,3131	0,041739	40,2969	1,1482	1414,1953
cg10071275	MYT1	0,1526	0,7107	0,5581	0,042185	478,6407	1,2434	184251,0423
cg26466858	LCE3A	0,1017	0,5238	0,4221	0,043295	2268,4921	1,2621	4077235,244
cg11847808	EPHA8	0,1216	0,4432	0,3215	0,043505	242,7398	1,1737	50203,9494
cg11102782	ISYNA1	0,1084	0,3385	0,2301	0,044452	116,1	1,1251	11980,2085
cg09462826	FADS1	0,1056	0,2969	0,1913	0,045115	1843,2617	1,1778	2884640,289
cg03876618	IGFBP7	0,1119	0,5243	0,4123	0,045289	142,2409	1,1096	18233,983
cg16954341	SCGN	0,209	0,4427	0,2337	0,045468	124,6317	1,1022	14092,9977
cg02780295	PCDHGC3	0,3671	0,4967	0,1296	0,045531	46,9832	1,0795	2044,7938
cg15901783	KCTD12	0,1389	0,5766	0,4377	0,046113	267,8732	1,1012	65161,0999
cg15835825	HTR5A	0,1801	0,3181	0,138	0,046348	173,5834	1,087	27718,4376
cg13936125	GNAO1	0,2468	0,3475	0,1007	0,046562	57,282	1,0636	3085,1244
cg25151295	RANBP5	0,1142	0,8162	0,702	0,047163	1613,4934	1,097	2373207,662
cg11465971	GSH2	0,3026	0,4333	0,1307	0,047296	48,4661	1,0474	2242,6485

cg25422943	PCDH9	0,4564	0,6115	0,1551	0,047309	30,8641	1,0416	914,5643
cg20066612	KCNJ9	0,1132	0,4164	0,3032	0,048428	385,5699	1,042	142675,7454
cg00176210	ANK1	0,1063	0,6375	0,5313	0,048579	2519,1235	1,0501	6043313,891
cg13216057	DKK3	0,2135	0,3684	0,1548	0,048638	55,0712	1,0242	2961,0417
cg27090216	TNFRSF10C	0,3483	0,5519	0,2035	0,048719	30,0173	1,0193	883,9819
cg00347904	SCUBE3	0,1581	0,6281	0,4699	0,049191	131,9908	1,0174	17122,8539
cg26450866	KCNA7	0,1585	0,3212	0,1627	0,049354	201,9234	1,0151	40165,7296
cg24454143	FLT3	0,1186	0,4467	0,3281	0,04976	180,2224	1,0055	32303,5328
cg24794531	TRPC1	0,1324	0,239	0,1066	0,049823	156,9391	1,0039	24533,6004
