# Supplementary material for: Multiple Sporadic Colorectal Cancers Display a Unique Methylation Phenotype
Source: PLoS One. 2014 Mar 18;9(3):e91033. doi: 10.1371/journal.pone.0091033 (PMC3958343; doi:10.1371/journal.pone.0091033)
Supplement: Table S4 — Hypermethylated CpG sites found in KRAS mutant versus. KRAS wild-type tumors based on the Infinium DNA methylation assay. (PDF) [file pone.0091033.s005.pdf]

Supplementary Table 4. Hypermethylated CpG sites found in *KRAS* mutant versus. *KRAS* wild-type tumors based on the Infinium DNA methylation assay.

TargetID	Symbol	beta.dif	beta.KRAS mutated	beta.KRAS wt	p	OR	ci.low	ci.high
cg14883392	ASCL1	0,1968	0,3583	0,1615	0,002795	193889391,9	714,3977	5,26221E+13
cg16778809	ADAM23	0,5142	0,6652	0,151	0,003026	398,0907	7,6097	20825,5314
cg22044848	RAFTLIN	0,3067	0,4189	0,1122	0,003612	235317,1833	56,7465	975816995,6
cg01717150	KCNK9	0,4211	0,5737	0,1526	0,003667	22470,7759	26,07	19368425,66
cg17791651	POU3F1	0,1872	0,6083	0,4211	0,004116	93806,3357	37,6341	233820791,6
cg22007439	DMRTA1	0,3585	0,4508	0,0923	0,004132	34092,5015	27,2253	42691846,06
cg23282559	KL	0,3629	0,5636	0,2007	0,004363	1188870,154	79,0915	17870596562
cg05714219	GALNT14	0,3224	0,6232	0,3008	0,004892	5962,3816	13,991	2540912,353
cg13168683	JAKMIP1	0,3454	0,5933	0,2479	0,005324	14438,1153	17,1409	12161516,89
cg07143898	SLIT1	0,2898	0,3955	0,1058	0,005493	4141,518	11,5842	1480656,458
cg15591678	ZNF365	0,3681	0,4645	0,0964	0,005701	8181,6058	13,7641	4863288,169
cg23166362	PIK3CD	0,3114	0,4454	0,134	0,006013	2882,8508	9,8018	847885,8509
cg08505473	PRIMA1	0,2802	0,4151	0,1349	0,006399	46738,4718	20,5591	106253973,1
cg17194182	EPO	0,2229	0,5375	0,3146	0,006633	2320,2632	8,624	624261,0498
cg09622447	CBS	0,2205	0,4268	0,2063	0,006671	25358,6541	16,6823	38547460,34
cg13818573	C1QL1	0,3658	0,6438	0,2779	0,006721	2412,8997	8,6395	673892,333
cg04172348	SYN2	0,2399	0,5485	0,3086	0,006812	2593,0495	8,7317	770057,9354
cg00263760	VAX1	0,2633	0,5415	0,2781	0,00703	3473,3575	9,249	1304385,349
cg15729869	BARHL1	0,1299	0,4177	0,2878	0,007228	1244498,813	44,4369	34853395595
cg04770504	DFNA5	0,1984	0,515	0,3165	0,007306	27276,8052	15,6619	47505489,79
cg01946574	PTPRM	0,2713	0,6748	0,4036	0,007632	31235,5626	15,5855	62600592,05
cg06230736	GATA3	0,4129	0,5632	0,1502	0,007645	175856,0208	24,6041	1256916417
cg18695917	FSTL5	0,17	0,288	0,118	0,007707	7313795,216	65,3537	8,18494E+11
cg26760212	GM632	0,1931	0,5663	0,3732	0,007771	245870,7046	26,3899	2290736778
cg24890043	DKFZP566N034	0,4831	0,6603	0,1772	0,007818	230,8587	4,1865	12730,4433
cg23473904	COL6A2	0,2718	0,4904	0,2186	0,00788	1535,7653	6,8558	344025,9785
cg27320127	KCNK12	0,1201	0,422	0,3019	0,007992	658837,6562	33,0412	13137164310
cg26258845	ADAM23	0,341	0,4934	0,1525	0,008663	1597,0364	6,4804	393574,2402
cg13297865	ELOVL4	0,4261	0,6164	0,1903	0,009066	519,3749	4,7419	56886,4287
cg22395019	GALNT14	0,2512	0,4267	0,1755	0,009379	1517,3007	6,0417	381050,9262
cg12374431	VAX2	0,3119	0,4356	0,1237	0,009519	1095,9376	5,5204	217571,59
cg24805239	DFNA5	0,1946	0,5499	0,3552	0,009698	24572,1216	11,5715	52179148,13
cg02545192	TERT	0,3629	0,4723	0,1093	0,009801	741,2723	4,9212	111657,5466
cg25657834	NTSR2	0,154	0,423	0,269	0,009813	2048652,36	33,2026	1,26405E+11
cg14147105	LOC93343	0,3248	0,4316	0,1068	0,010098	15274,6375	9,913	23536210,48
cg08380205	SPSB4	0,2368	0,5653	0,3285	0,01018	10796,7804	9,0562	12871937,68
cg07112210	MGAT5B	0,123	0,4661	0,3431	0,010221	1605183,9	29,4969	87352052343
cg21321735	KIF1A	0,2268	0,3285	0,1016	0,010277	7266,4507	8,1734	6460150,812

cg11452221	GEFT	0,2181	0,5272	0,3091	0,010416	47916,2873	12,5727	182615872,9
cg07075930	PTPRM	0,2493	0,4194	0,1701	0,0105	1420,8333	5,4688	369144,9041
cg14242042	SOX5	0,3826	0,486	0,1034	0,010507	150,5295	3,2327	7009,4462
cg04836786	SMARCA3	0,4908	0,5837	0,093	0,01051	97,437	2,9194	3252,0315
cg24778383	TNFSF7	0,3153	0,5011	0,1859	0,010721	935,6827	4,8863	179176,1641
cg09450238	BTBD6	0,1694	0,5588	0,3894	0,010951	1893752,348	27,6451	1,29726E+11
cg17834752	KCNK9	0,1586	0,5954	0,4368	0,011107	9146917,654	38,7447	2,15942E+12
cg21601405	CD40	0,2611	0,626	0,3648	0,011118	43836,3191	11,4436	167921360,8
cg22268231	SPIB	0,1667	0,4437	0,2771	0,011481	2201497,432	26,5858	1,823E+11
cg03853987	CHST10	0,239	0,7733	0,5344	0,011516	558,0531	4,1307	75392,8012
cg15107670	WT1	0,2271	0,6243	0,3972	0,011616	3413,9782	6,1536	1894053,538
cg21937886	GFPT2	0,3237	0,6775	0,3538	0,011986	543,5668	3,9955	73948,8342
cg25302419	CTNND2	0,3474	0,6213	0,2738	0,012077	566,7496	4,0113	80075,8114
cg19764436	GNAZ	0,1737	0,4687	0,295	0,012146	13653,7054	8,0067	23283365,44
cg01519742	JAKMIP1	0,2431	0,653	0,4098	0,012222	470,8035	3,8205	58017,945
cg10762615	FBXW10	0,1291	0,7803	0,6512	0,012396	824548,2144	19,02	35745562861
cg18995088	CXCL14	0,2963	0,4618	0,1655	0,012777	299,1124	3,3648	26589,2031
cg25971347	FOXF1	0,1766	0,5792	0,4025	0,013524	1865,9247	4,7322	735734,0749
cg03593419	GABRA4	0,2204	0,3226	0,1023	0,013686	12096,38	6,8707	21296493,9
cg17503456	FOXL2	0,1951	0,3279	0,1327	0,01385	1748,1445	4,5741	668116,5336
cg22036988	SPSB4	0,316	0,7011	0,3851	0,013889	10112542,84	26,5542	3,85113E+12
cg00107187	FLJ42486	0,1515	0,619	0,4675	0,014035	530257,2119	14,3506	19593094362
cg13801416	AKR1B1	0,172	0,699	0,527	0,014309	229,9147	2,9642	17833,3214
cg19528976	FLJ39237	0,1881	0,427	0,2389	0,014483	5393,4258	5,5005	5288401,445
cg19286604	MGC39545	0,2407	0,4449	0,2042	0,014884	5190,1366	5,3092	5073744,123
cg23040064	JPH3	0,2259	0,5148	0,2889	0,015069	2759,1832	4,6385	1641284,925
cg07651242	ADCY1	0,3091	0,7354	0,4263	0,015161	2213,2275	4,4196	1108321,931
cg18938204	EMILIN3	0,1637	0,6328	0,4692	0,015638	1999,106	4,2115	948923,456
cg17414107	GNAS	0,1265	0,6559	0,5295	0,01587	1696059,343	14,6977	1,95719E+11
cg08319991	UCHL1	0,2799	0,5019	0,222	0,01597	315,536	2,9264	34022,3602
cg24813212	TEKT2	0,2562	0,4058	0,1497	0,016293	18425,2089	6,1007	55647636,41
cg13226172	CNTFR	0,1553	0,3238	0,1684	0,016374	20545,9216	6,1861	68239488,9
cg22594309	SYT2	0,3011	0,5923	0,2912	0,016899	224,5101	2,6435	19067,4099
cg02600430	ZIC5	0,1014	0,3943	0,2929	0,017024	39594,7907	6,6259	236610536,1
cg22026853	POU3F2	0,2531	0,3946	0,1415	0,017104	400,4923	2,9063	55189,1184
cg06557358	LOC124842	0,1733	0,5862	0,4129	0,017229	15273,4529	5,508	42352867,46
cg25808906	DGKG	0,3215	0,4696	0,1482	0,017245	350,4129	2,8206	43532,5604
cg07641524	MSRB3	0,3675	0,5485	0,181	0,017299	128,7079	2,3579	7025,6316
cg04603031	CHRNA3	0,294	0,6167	0,3227	0,017404	220,5933	2,5825	18843,0092
cg07017374	FLT3	0,3904	0,7116	0,3213	0,017477	4596,6547	4,3847	4818855,611
cg18821742	PCDH7	0,1825	0,5179	0,3354	0,017492	942,5799	3,3189	267692,2055
cg13449778	C1orf76	0,1275	0,5961	0,4687	0,017523	4204,2431	4,3047	4106122,607
cg09660171	LMX1B	0,1916	0,418	0,2264	0,017628	24993,8327	5,8344	107070512,6
cg14356550	KLHDC7A	0,1186	0,6073	0,4887	0,018422	400404,2983	8,7864	18246891682
cg06781209	FADS2	0,1465	0,5549	0,4084	0,018434	2024,2325	3,6031	1137213,882

cg08820801	FBXO17	0,1916	0,7014	0,5098	0,018594	76325,794	6,5549	888740989,8
cg13686115	PHYHIPL	0,1631	0,3328	0,1697	0,018674	10741,1012	4,6975	24560116,69
cg02361557	NTNG1	0,3481	0,4839	0,1359	0,018733	290,4153	2,5674	32851,1907
cg20804555	GSC	0,1895	0,5158	0,3263	0,018875	1534,3697	3,3612	700430,3286
cg26450866	KCNA7	0,164	0,3212	0,1572	0,018999	6284,1517	4,2111	9377762,952
cg26717133	PCDH21	0,1083	0,7093	0,601	0,019197	2038922,858	10,6788	3,89297E+11
cg01920829	CDK5R2	0,2192	0,5389	0,3197	0,019608	1660,1487	3,2792	840465,4267
cg27661264	GNAS	0,1411	0,5786	0,4375	0,019703	29133,7708	5,1542	164675164,1
cg00318573	CHRNA4	0,113	0,6326	0,5196	0,019867	330256,4901	7,485	14571759200
cg21553524	MGC33926	0,3304	0,438	0,1077	0,019987	387,4668	2,5577	58697,085
cg07845566	B3GAT1	0,1653	0,2498	0,0845	0,020414	344,6886	2,469	48119,9769
cg09945801	WRN	0,1294	0,5979	0,4685	0,021024	3458,5568	3,4125	3505277,159
cg26776069	C1orf176	0,1146	0,1821	0,0675	0,021133	1430,1181	2,9718	688212,7167
cg02508567	TCF7L1	0,2254	0,5418	0,3165	0,022184	227,0776	2,1732	23726,9606
cg13666729	ZBTB8	0,1988	0,4269	0,2281	0,02221	404,3022	2,3579	69324,7317
cg11177693	ZNF513	0,16	0,5368	0,3768	0,022966	9408,9592	3,5379	25022575,41
cg09276363	KCND3	0,1008	0,4633	0,3625	0,023099	38098,2944	4,2539	341213028,2
cg08453021	ELMO1	0,1332	0,5986	0,4653	0,023103	674,8212	2,4449	186259,6412
cg08315770	KCNK17	0,2569	0,4511	0,1942	0,023286	200,8918	2,0578	19611,8126
cg00603172	BOK	0,119	0,7121	0,5932	0,02337	68462,4698	4,5239	1036071930
cg10919204	CDH6	0,1044	0,6051	0,5007	0,02338	48550,3063	4,3149	546278022,3
cg06817264	CTSF	0,1125	0,6709	0,5584	0,023401	43112,6756	4,2403	438338830,8
cg10920957	JPH3	0,2657	0,4542	0,1884	0,024165	201,7983	2,0004	20356,8597
cg14662379	KIF1A	0,2261	0,5836	0,3575	0,024318	462,34	2,2164	96443,5028
cg19674669	LOC112937	0,1146	0,6757	0,5612	0,024324	5616,308	3,0633	10297027,12
cg19118812	ELMO1	0,204	0,6546	0,4507	0,024343	93,7057	1,8007	4876,2428
cg15042080	TMEM74	0,3943	0,5154	0,1212	0,024359	98,2949	1,8111	5334,9006
cg03171924	RUNX3	0,1299	0,7316	0,6016	0,02445	100603,4238	4,414	2292962603
cg14186992	HKR3	0,131	0,8444	0,7134	0,024615	15401,0794	3,4322	69108584,71
cg15239123	SMOC1	0,3929	0,5377	0,1449	0,024764	109,4181	1,8153	6595,3787
cg17775235	NPTX1	0,2014	0,3582	0,1568	0,025046	188,6241	1,928	18453,9495
cg13481359	EGR4	0,1161	0,5561	0,44	0,025169	4392,0664	2,8423	6786780,196
cg09979256	RSPO3	0,2961	0,4898	0,1937	0,025222	336,7298	2,0605	55029,6073
cg26702254	KCND2	0,1134	0,4334	0,3201	0,025266	670,4872	2,2407	200633,2437
cg01313514	WNT3A	0,2072	0,4832	0,276	0,025416	1298,119	2,4163	697395,8885
cg02503850	ADAMTS14	0,1833	0,5955	0,4122	0,025756	204,8507	1,9045	22034,3992
cg12858460	EOMES	0,2066	0,3366	0,1299	0,025788	3005,7878	2,6322	3432458,317
cg15057581	PTPNS1	0,1918	0,3024	0,1105	0,026363	1464,8755	2,3542	911487,0787
cg20881054	VASH1	0,1568	0,2415	0,0847	0,02639	1162313,276	5,1459	2,62535E+11
cg22156632	WNT6	0,1347	0,4729	0,3383	0,026405	6424,2283	2,7947	14767410,74
cg24250393	PRKCB1	0,1995	0,4395	0,2399	0,02666	452,5621	2,0292	100933,4854
cg08853659	CLSTN2	0,3007	0,4323	0,1316	0,026839	72,4897	1,6343	3215,2352
cg20648149	SYNE2	0,106	0,7236	0,6176	0,026844	520156,9176	4,5223	59828857049
cg00625653	WNT7A	0,2204	0,4672	0,2468	0,027032	124,8665	1,7301	9011,9652
cg08056146	SOX7	0,4964	0,6286	0,1322	0,027533	34,5801	1,4802	807,8766

cg08573687	TH	0,1096	0,8944	0,7848	0,027894	622665,7632	4,2589	91034966847
cg00826384	PTPRZ1	0,3069	0,4681	0,1612	0,028464	1160,0225	2,1035	639733,8633
cg12420104	DMRT3	0,3056	0,5613	0,2558	0,028733	81,5972	1,5796	4214,9742
cg07307078	TUBB6	0,3198	0,6377	0,3178	0,02901	243,8364	1,7548	33881,9236
cg01757745	C10orf93	0,1295	0,5632	0,4337	0,029513	7293,1114	2,4234	21948407,57
cg14348532	CALCA	0,1048	0,3278	0,223	0,029886	19880,4221	2,6237	150641142
cg18952560	PTPNS1	0,1213	0,2521	0,1308	0,030448	1551,1466	2,0004	1202792,851
cg14167596	MSX1	0,1265	0,347	0,2206	0,030848	207233,1595	3,0915	13891465437
cg00654814	MGAT5B	0,2333	0,6761	0,4429	0,030958	287,9898	1,6799	49369,7232
cg10249734	SECTM1	0,1083	0,5563	0,448	0,031558	20025,9133	2,3997	167118568,4
cg26646370	SHD	0,19	0,3762	0,1862	0,031996	82,149	1,4612	4618,2926
cg10364513	RXRG	0,1427	0,5325	0,3899	0,032486	449,5863	1,6647	121421,6884
cg10158080	SOX5	0,1645	0,3021	0,1376	0,032938	104,3848	1,4575	7475,7103
cg22633722	CBS	0,2068	0,4429	0,2361	0,032995	502,0112	1,6523	152521,1262
cg10979891	SMOC1	0,2424	0,5573	0,315	0,033067	207,8928	1,5357	28143,4821
cg17606785	EFS	0,1274	0,5067	0,3793	0,03457	2067,5164	1,7404	2456077,362
cg18602314	GFPT2	0,1744	0,3403	0,1659	0,034865	324,5062	1,5083	69817,8586
cg09260441	DOK5	0,174	0,5315	0,3575	0,034934	166,7455	1,436	19362,5254
cg04807655	TIAM1	0,423	0,6344	0,2114	0,035076	125,5708	1,4025	11242,5533
cg18946226	MYR8	0,1072	0,5969	0,4896	0,035114	5666,3376	1,8281	17563256,85
cg17162024	UNQ9433	0,2747	0,6913	0,4166	0,035922	42,6784	1,2797	1423,2986
cg05203877	SLC8A3	0,3205	0,5283	0,2078	0,036145	146,4055	1,38	15532,7247
cg00943909	GNAS	0,1172	0,7314	0,6142	0,036208	234027,2591	2,2135	24742831713
cg04525496	CSPG2	0,1052	0,3332	0,228	0,036929	1460,7754	1,5559	1371457,949
cg18123948	GATA4	0,2803	0,4784	0,1982	0,037111	138,6716	1,3428	14320,8281
cg23917399	TNFAIP8	0,3537	0,5364	0,1827	0,037165	54,9636	1,2692	2380,2469
cg08274234	SEMA3E	0,1231	0,4964	0,3733	0,037643	569,3156	1,4369	225573,9493
cg06905514	CAMK2B	0,1564	0,6239	0,4675	0,037702	79,6123	1,2825	4942,1264
cg07109287	LHX2	0,3137	0,5801	0,2664	0,038353	107,9507	1,2855	9065,303
cg01775414	PHF21B	0,2129	0,6474	0,4345	0,038715	170,692	1,3056	22316,6053
cg03819692	FLJ36701	0,1385	0,7428	0,6043	0,040512	1077,4378	1,3522	858505,9703
cg07015629	ERBB4	0,2123	0,6454	0,4331	0,040512	70,4186	1,2018	4126,0091
cg03483654	DAK	0,1975	0,8815	0,684	0,040746	298,4594	1,2711	70076,8983
cg11563860	CNTN4	0,1506	0,4699	0,3194	0,040808	716,3574	1,3163	389856,4017
cg26884581	PYGM	0,1131	0,6107	0,4976	0,040856	13742,8889	1,4861	127090308,3
cg25422943	PCDH9	0,4987	0,6125	0,1138	0,041455	43,1574	1,157	1609,8048
cg05497616	CAMK4	0,1444	0,2298	0,0854	0,041616	464,1747	1,2626	170640,1714
cg12867448	ODF2	0,1216	0,7589	0,6374	0,041726	20553,196	1,4506	291203334,1
cg09495977	HTRA3	0,1047	0,4422	0,3376	0,041842	1025,2075	1,2916	813739,7069
cg25549459	POU3F3	0,128	0,2932	0,1652	0,042071	931,2883	1,2777	678821,8435
cg22231902	EN1	0,3177	0,5247	0,207	0,042116	106,5148	1,1809	9607,1069
cg14046986	RCSD1	0,1164	0,1944	0,078	0,042123	484,9159	1,2462	188686,3683
cg14312526	FOXL2	0,1875	0,3822	0,1947	0,042133	134,1055	1,1902	15110,0919
cg00687686	NDRG4	0,1088	0,6388	0,53	0,042226	117,1999	1,1821	11620,2156
cg05028467	SNCB	0,2319	0,5397	0,3078	0,043096	100,011	1,1538	8669,2745

cg09874752	SFRP5	0,2322	0,4205	0,1884	0,043519	59,763	1,1264	3170,9108
cg16773899	EDIL3	0,1889	0,5692	0,3802	0,043961	117,9635	1,1378	12230,459
cg18581445	SHD	0,1343	0,3279	0,1936	0,044241	268,7926	1,155	62552,2015
cg25157874	QKI	0,3234	0,56	0,2367	0,044382	33,7312	1,0924	1041,5459
cg21835643	RBPSUHL	0,2677	0,6686	0,4009	0,044512	260,0137	1,1461	58987,9504
cg01295203	PRDM14	0,125	0,7429	0,6179	0,045149	229,4732	1,1247	46821,0596
cg15014549	FLJ10847	0,1494	0,2753	0,1259	0,045418	127,5695	1,1039	14741,9706
cg19461621	COLEC12	0,1291	0,6377	0,5086	0,045463	947,0005	1,1484	780934,4414
cg13749822	HHIP	0,1309	0,5195	0,3886	0,046012	360,3946	1,1098	117029,7192
cg17183546	D4S234E	0,1004	0,4943	0,3939	0,046078	617,9325	1,1183	341433,4278
cg26021627	UNQ9433	0,2322	0,5517	0,3195	0,047135	85,7898	1,0579	6956,7516
cg04274487	RAB31	0,1461	0,2376	0,0915	0,047295	162,1274	1,0626	24735,9963
cg00565688	TP73	0,3046	0,6017	0,2972	0,047387	113,6536	1,0561	12231,154
cg26270746	GSH1	0,181	0,4548	0,2738	0,0481	339,1604	1,0499	109561,6387
cg24019564	RUNX3	0,1508	0,6357	0,4849	0,048758	1051,0657	1,0386	1063653,316
cg04969808	WNT7A	0,1296	0,4151	0,2855	0,04933	62,9787	1,0122	3918,4538
