# Supplementary material for: Multiple Sporadic Colorectal Cancers Display a Unique Methylation Phenotype
Source: PLoS One. 2014 Mar 18;9(3):e91033. doi: 10.1371/journal.pone.0091033 (PMC3958343; doi:10.1371/journal.pone.0091033)
Supplement: Table S5 — Functional annotation clustering of differentially methylated genes found in multiple versus solitary tumors based on DAVID analysis. (PDF) [file pone.0091033.s006.pdf]

Category	Term	Count	PValue	Genes
GOTERM_BP_FAT	GO:0030182~neuron differentiation	14	1,54103E-07	RET, TUBB2B, GDF7, ERBB2, MAP1B, DPYSL5, SOX5, CXCL12, GLI3, SMO, UNC5A, DLX5, GBX2, RUNX3
GOTERM_BP_FAT	GO:0048812~neuron projection morphogenesis	10	8,54455E-07	SMO, GDF7, UNC5A, ERBB2, DLX5, MAP1B, GBX2, DPYSL5, CXCL12, RUNX3
GOTERM_BP_FAT	GO:0001667~ameboidal cell migration	6	9,15386E-07	FGF19, SMO, RET, GBX2, KITLG, CXCL12
GOTERM_BP_FAT	GO:0048858~cell projection morphogenesis	10	2,72563E-06	SMO, GDF7, UNC5A, ERBB2, DLX5, MAP1B, GBX2, DPYSL5, CXCL12, RUNX3
GOTERM_BP_FAT	GO:0032990~cell part morphogenesis	10	3,90539E-06	SMO, GDF7, UNC5A, ERBB2, DLX5, MAP1B, GBX2, DPYSL5, CXCL12, RUNX3
GOTERM_BP_FAT	GO:0031175~neuron projection development	10	3,90539E-06	SMO, GDF7, UNC5A, ERBB2, DLX5, MAP1B, GBX2, DPYSL5, CXCL12, RUNX3
GOTERM_BP_FAT	GO:0007409~axonogenesis	9	4,20155E-06	GDF7, UNC5A, ERBB2, DLX5, MAP1B, GBX2, DPYSL5, CXCL12, RUNX3
GOTERM_BP_FAT	GO:0001755~neural crest cell migration	5	4,26708E-06	FGF19, SMO, RET, GBX2, KITLG
GOTERM_BP_FAT	GO:0048666~neuron development	11	5,18832E-06	SMO, RET, GDF7, UNC5A, ERBB2, DLX5, MAP1B, GBX2, DPYSL5, CXCL12, RUNX3
GOTERM_BP_FAT	GO:0048667~cell morphogenesis involved in neuron differentiation	9	7,55866E-06	GDF7, UNC5A, ERBB2, DLX5, MAP1B, GBX2, DPYSL5, CXCL12, RUNX3
SP_PIR_KEYWORDS	glycoprotein	37	8,94936E-06	CLSTN2, SLC15A1, GDF7, FAM20A, ERBB2, ADCY5, KITLG, ASTN2, MEGF11, MEGF10, CPZ, HRH3, RSPO1, UNC5A, PGLYRP2, HS6ST3, PDGFC, HCN4, HTR5A, RET, ST6GAL2, CCKBR, HTR4, BSN, PCDH9, B3GAT2, SMO, TNFRSF10C, ACCN4, LASS1, WNT9B, CACNA1G, CACNA1H, KCNH2, ABCC8, IGFBP3, LRP3
GOTERM_BP_FAT	GO:0007411~axon guidance	7	1,31461E-05	GDF7, UNC5A, ERBB2, GBX2, DPYSL5, CXCL12, RUNX3
GOTERM_BP_FAT	GO:0006928~cell motion	12	1,66821E-05	FGF19, SMO, RET, GDF7, ARHGEF7, UNC5A, ERBB2, GBX2, DPYSL5, KITLG, CXCL12, RUNX3
GOTERM_BP_FAT	GO:0014033~neural crest cell differentiation	5	1,90089E-05	FGF19, SMO, RET, GBX2, KITLG
GOTERM_BP_FAT	GO:0014032~neural crest cell development	5	1,90089E-05	FGF19, SMO, RET, GBX2, KITLG
GOTERM_BP_FAT	GO:0000904~cell morphogenesis involved in differentiation	9	2,32635E-05	GDF7, UNC5A, ERBB2, DLX5, MAP1B, GBX2, DPYSL5, CXCL12, RUNX3
UP_SEQ_FEATURE	glycosylation site:N-linked (GlcNAc,,,)	35	2,89804E-05	CLSTN2, SLC15A1, GDF7, FAM20A, ERBB2, ADCY5, KITLG, ASTN2, MEGF11, MEGF10, CPZ, RSPO1, HRH3, UNC5A, PGLYRP2, HS6ST3, PDGFC, HCN4, HTR5A, RET, ST6GAL2, CCKBR, HTR4, PCDH9, B3GAT2, SMO, ACCN4, LASS1, WNT9B, CACNA1G, CACNA1H, KCNH2, ABCC8, IGFBP3, LRP3
GOTERM_BP_FAT	GO:0000902~cell morphogenesis	10	5,39191E-05	SMO, GDF7, UNC5A, ERBB2, DLX5, MAP1B, GBX2, DPYSL5, CXCL12, RUNX3
GOTERM_BP_FAT	GO:0030030~cell projection organization	10	6,96308E-05	SMO, GDF7, UNC5A, ERBB2, DLX5, MAP1B, GBX2, DPYSL5, CXCL12, RUNX3
GOTERM_BP_FAT	GO:0048483~autonomic nervous system development	4	0,000101641	SMO, RET, ERBB2, GBX2
GOTERM_BP_FAT	GO:0014031~mesenchymal cell development	5	0,000108684	FGF19, SMO, RET, GBX2, KITLG
GOTERM_BP_FAT	GO:0048762~mesenchymal cell differentiation	5	0,000108684	FGF19, SMO, RET, GBX2, KITLG
GOTERM_BP_FAT	GO:0060485~mesenchyme development	5	0,000117311	FGF19, SMO, RET, GBX2, KITLG
GOTERM_BP_FAT	GO:0045597~positive regulation of cell differentiation	8	0,000121128	SMO, IRX3, THRB, DLX5, MAP1B, KITLG, IGFBP3, GLI3
GOTERM_BP_FAT	GO:0032989~cellular component morphogenesis	10	0,000124206	SMO, GDF7, UNC5A, ERBB2, DLX5, MAP1B, GBX2, DPYSL5, CXCL12, RUNX3
SP_PIR_KEYWORDS	growth factor	6	0,000229759	FGF19, GDF7, LASS1, KITLG, PDGFC, CXCL12
GOTERM_BP_FAT	GO:0050678~regulation of epithelial cell proliferation	5	0,000392792	HRH3, ERBB2, DLX5, FOXE3, RUNX3
GOTERM_BP_FAT	GO:0051094~positive regulation of developmental process	8	0,000396727	SMO, IRX3, THRB, DLX5, MAP1B, KITLG, IGFBP3, GLI3
GOTERM_BP_FAT	GO:0008016~regulation of heart contraction	5	0,000535225	THRB, CACNA1G, CACNA1H, HCN4, KCNH2
SP_PIR_KEYWORDS	signal	27	0,000583332	FGF19, CLSTN2, GDF7, FAM20A, ERBB2, KITLG, ASTN2, MEGF11, MEGF10, CXCL12, TIMP3, CPZ, RSPO1, UNC5A, HTRA1, PGLYRP2, PDGFC, GPX7, COL8A1, RET, PCDH9, SMO, TNFRSF10C, LASS1, WNT9B, IGFBP3, LRP3
UP_SEQ_FEATURE	signal peptide	27	0,000644198	FGF19, CLSTN2, GDF7, FAM20A, ERBB2, KITLG, ASTN2, MEGF11, MEGF10, CXCL12, TIMP3, CPZ, RSPO1, UNC5A, HTRA1, PGLYRP2, PDGFC, GPX7, COL8A1, RET, PCDH9, SMO, TNFRSF10C, LASS1, WNT9B, IGFBP3, LRP3
GOTERM_BP_FAT	GO:0001501~skeletal system development	8	0,000897323	SMO, ALOX15, DLX5, SOX5, TFAP2A, ALX4, IGFBP3, GLI3
GOTERM_MF_FAT	GO:0003700~transcription factor activity	14	0,000934574	IRX3, THRB, SOX14, SOX5, ZNF232, SOX8, GLI3, DLX5, GBX2, HIF3A, TFAP2A, ALX4, RUNX3, FOXE3
GOTERM_BP_FAT	GO:0050679~positive regulation of epithelial cell proliferation	4	0,001035678	HRH3, ERBB2, DLX5, FOXE3
UP_SEQ_FEATURE	disulfide bond	24	0,001135795	FGF19, ST6GAL2, CCKBR, GDF7, ERBB2, HTR4, KITLG, ASTN2, MEGF11, TIMP3, CXCL12, MEGF10, CPZ, SMO, ACCN4, TNFRSF10C, RSPO1, UNC5A, PGLYRP2, LASS1, PDGFC, IGFBP3, LRP3, HTR5A
UP_SEQ_FEATURE	topological domain:Cytoplasmic	27	0,001144859	CLSTN2, SLC15A1, ERBB2, ADCY5, CABP7, KITLG, ASTN2, MEGF10, HRH3, UNC5A, HS6ST3, SLC30A2, HCN4, HTR5A, RET, ST6GAL2, CCKBR, HTR4, PCDH9, B3GAT2, SMO, ACCN4, CACNA1G, CACNA1H, KCNH2, LRP3, ABCC8
GOTERM_MF_FAT	GO:0008083~growth factor activity	6	0,001249795	FGF19, GDF7, LASS1, KITLG, PDGFC, CXCL12
SP_PIR_KEYWORDS	disulfide bond	24	0,001714715	FGF19, ST6GAL2, CCKBR, GDF7, ERBB2, HTR4, KITLG, ASTN2, MEGF11, TIMP3, CXCL12, MEGF10, CPZ, SMO, ACCN4, TNFRSF10C, RSPO1, UNC5A, PGLYRP2, LASS1, PDGFC, IGFBP3, LRP3, HTR5A
SP_PIR_KEYWORDS	dna-binding	18	0,001748036	IRX3, MTERF, ZNF264, THRB, SOX14, SOX5, ZNF232, PRDM16, SOX8, GLI3, DLX5, ZNF681, HIF3A, GBX2, TFAP2A, ALX4, RUNX3, FOXE3
GOTERM_BP_FAT	GO:0016477~cell migration	7	0,002226607	FGF19, SMO, RET, ARHGEF7, GBX2, KITLG, CXCL12
KEGG_PATHWAY	hsa05200:Pathways in cancer	8	0,002975368	FGF19, SMO, RET, ERBB2, WNT9B, KITLG, GLI3, DAPK1
GOTERM_BP_FAT	GO:0035239~tube morphogenesis	5	0,003407516	RET, GBX2, TFAP2A, CXCL12, GLI3
GOTERM_BP_FAT	GO:0051674~localization of cell	7	0,003771569	FGF19, SMO, RET, ARHGEF7, GBX2, KITLG, CXCL12
GOTERM_BP_FAT	GO:0048870~cell motility	7	0,003771569	FGF19, SMO, RET, ARHGEF7, GBX2, KITLG, CXCL12
KEGG_PATHWAY	hsa04020:Calcium signaling pathway	6	0,003836019	CCKBR, ERBB2, HTR4, CACNA1G, CACNA1H, HTR5A
GOTERM_BP_FAT	GO:0008284~positive regulation of cell proliferation	8	0,003931603	SMO, HRH3, CCKBR, ERBB2, DLX5, KITLG, PDGFC, FOXE3
GOTERM_BP_FAT	GO:0048562~embryonic organ morphogenesis	5	0,004019026	DLX5, GBX2, TFAP2A, ALX4, GLI3
SP_PIR_KEYWORDS	Secreted	16	0,004261695	FGF19, GDF7, FAM20A, KITLG, CXCL12, TIMP3, CPZ, RSPO1, HTRA1, PGLYRP2, LASS1, WNT9B, PDGFC, GPX7, COL8A1, IGFBP3
GOTERM_BP_FAT	GO:0021915~neural tube development	4	0,004433906	SMO, GBX2, TFAP2A, GLI3
GOTERM_BP_FAT	GO:0042127~regulation of cell proliferation	11	0,004572205	SMO, HRH3, CCKBR, ERBB2, DLX5, KITLG, PDGFC, IGFBP3, GLI3, FOXE3, RUNX3
GOTERM_CC_FAT	GO:0044421~extracellular region part	12	0,004599281	RSPO1, GDF7, HTRA1, LASS1, WNT9B, KITLG, PDGFC, COL8A1, IGFBP3, TIMP3, CXCL12, CPZ
GOTERM_BP_FAT	GO:0045165~cell fate commitment	5	0,004700989	SMO, GDF7, SOX5, SOX8, GLI3
GOTERM_BP_FAT	GO:0007423~sensory organ development	6	0,005143071	ERBB2, DLX5, GBX2, COL8A1, GLI3, FOXE3
GOTERM_BP_FAT	GO:0043009~chordate embryonic development	7	0,005430578	SMO, GBX2, TFAP2A, HCN4, ALX4, SOX8, GLI3
GOTERM_BP_FAT	GO:0009792~embryonic development ending in birth or egg hatching	7	0,005670447	SMO, GBX2, TFAP2A, HCN4, ALX4, SOX8, GLI3
GOTERM_BP_FAT	GO:0019932~second-messenger-mediated signaling	6	0,005730465	HRH3, CCKBR, ADCY5, ERBB2, HTR4, HTR5A
GOTERM_MF_FAT	GO:0046873~metal ion transmembrane transporter activity	7	0,005868308	ACCN4, CACNA1G, CACNA1H, SLC30A2, HCN4, KCNH2, ABCC8
GOTERM_MF_FAT	GO:0030528~transcription regulator activity	16	0,006749854	IRX3, MTERF, THRB, SOX14, SOX5, ZNF232, PRDM16, SOX8, GLI3, DLX5, GBX2, HIF3A, TFAP2A, ALX4, RUNX3, FOXE3
GOTERM_CC_FAT	GO:0043025~cell soma	5	0,007928727	SMO, MAP1B, CACNA1G, DPYSL5, CACNA1H
UP_SEQ_FEATURE	topological domain:Extracellular	21	0,008486571	RET, CLSTN2, SLC15A1, CCKBR, ERBB2, ADCY5, HTR4, KITLG, PCDH9, CABP7, ASTN2, MEGF10, SMO, ACCN4, HRH3, UNC5A, CACNA1G, CACNA1H, ABCC8, LRP3, HTR5A
SP_PIR_KEYWORDS	transcription regulation	17	0,009600471	IRX3, MTERF, ZNF264, THRB, SOX14, SOX5, ZNF232, PRDM16, SOX8, GLI3, ZNF681, GBX2, HIF3A, TFAP2A, ALX4, RUNX3, FOXE3
GOTERM_BP_FAT	GO:0048568~embryonic organ development	5	0,009864373	DLX5, GBX2, TFAP2A, ALX4, GLI3
GOTERM_MF_FAT	GO:0043565~sequence-specific DNA binding	9	0,010501237	IRX3, THRB, SOX14, DLX5, GBX2, PRDM16, ALX4, GLI3, FOXE3
GOTERM_BP_FAT	GO:0045935~positive regulation of nucleobase, nucleoside, nucleotide and nucleic acid metabolic process	9	0,010642558	THRB, GDF7, SOX5, KITLG, PDGFC, PRDM16, ALX4, SOX8, GLI3
SP_PIR_KEYWORDS	Transcription	17	0,011724073	IRX3, MTERF, ZNF264, THRB, SOX14, SOX5, ZNF232, PRDM16, SOX8, GLI3, ZNF681, GBX2, HIF3A, TFAP2A, ALX4, RUNX3, FOXE3
GOTERM_BP_FAT	GO:0040007~growth	5	0,012174566	SMO, ST6GAL2, GDF7, TIMP3, GLI3

GOTERM_BP_FAT	GO:0051173~positive regulation of nitrogen compound metabolic process	9	0,012705889	THRB, GDF7, SOX5, KITLG, PDGFC, PRDM16, ALX4, SOX8, GLI3
GOTERM_BP_FAT	GO:0008015~blood circulation	5	0,012859933	HRH3, CACNA1G, HCN4, KCNH2, CXCL12
GOTERM_BP_FAT	GO:0003013~circulatory system process	5	0,012859933	HRH3, CACNA1G, HCN4, KCNH2, CXCL12
GOTERM_BP_FAT	GO:0021700~developmental maturation	4	0,013158637	RET, MAP1B, SOX8, RUNX3
INTERPRO	IPR005445:Voltage-dependent calcium channel, T-type, alpha-1 subunit	2	0,013624767	CACNA1G, CACNA1H
GOTERM_BP_FAT	GO:0010557~positive regulation of macromolecule biosynthetic process	9	0,013842177	THRB, GDF7, SOX5, KITLG, PDGFC, PRDM16, ALX4, SOX8, GLI3
GOTERM_BP_FAT	GO:0006351~transcription, DNA-dependent	6	0,013855605	MTERF, THRB, HIF3A, SOX5, FOXE3, RUNX3
INTERPRO	IPR005821:Ion transport	4	0,013892616	CACNA1G, CACNA1H, HCN4, KCNH2
GOTERM_BP_FAT	GO:0021910~smoothened signaling pathway involved in ventral spinal cord patterning	2	0,01456609	SMO, GLI3
GOTERM_BP_FAT	GO:0032774~RNA biosynthetic process	6	0,01461999	MTERF, THRB, HIF3A, SOX5, FOXE3, RUNX3
GOTERM_MF_FAT	GO:0008332~low voltage-gated calcium channel activity	2	0,014945715	CACNA1G, CACNA1H
UP_SEQ_FEATURE	DNA-binding region:HMG box	3	0,01495596	SOX14, SOX5, SOX8
GOTERM_CC_FAT	GO:0042995~cell projection	9	0,015631578	SMO, ARHGEF7, ERBB2, MAP1B, CACNA1G, DPYSL5, CACNA1H, HCN4, GLI3
UP_SEQ_FEATURE	transmembrane region	31	0,015783283	CYB5R1, CLSTN2, SLC15A1, ERBB2, ADCY5, CABP7, KITLG, ASTN2, MEGF11, MEGF10, HRH3, UNC5A, HS6ST3, SLC30A2, HCN4, HTR5A, RET, ST6GAL2, CCKBR, HTR4, PCDH9, UCP1, B3GAT2, SMO, ACCN4, LASS1, CACNA1G, CACNA1H, KCNH2, ABCC8, LRP3
GOTERM_BP_FAT	GO:0048598~embryonic morphogenesis	6	0,016867529	RET, DLX5, GBX2, TFAP2A, ALX4, GLI3
SP_PIR_KEYWORDS	transmembrane	31	0,017190163	CYB5R1, CLSTN2, SLC15A1, ERBB2, ADCY5, CABP7, KITLG, ASTN2, MEGF11, MEGF10, HRH3, UNC5A, HS6ST3, SLC30A2, HCN4, HTR5A, RET, ST6GAL2, CCKBR, HTR4, PCDH9, UCP1, B3GAT2, SMO, ACCN4, LASS1, CACNA1G, CACNA1H, KCNH2, ABCC8, LRP3
GOTERM_BP_FAT	GO:0044057~regulation of system process	6	0,01729957	THRB, MAP1B, CACNA1G, CACNA1H, HCN4, KCNH2
GOTERM_BP_FAT	GO:0060284~regulation of cell development	5	0,017775598	SMO, IRX3, MAP1B, SOX5, IGFBP3
GOTERM_BP_FAT	GO:0006812~cation transport	8	0,017800608	ACCN4, CACNA1G, CACNA1H, SLC30A2, UCP1, HCN4, KCNH2, ABCC8
GOTERM_BP_FAT	GO:0031328~positive regulation of cellular biosynthetic process	9	0,017841452	THRB, GDF7, SOX5, KITLG, PDGFC, PRDM16, ALX4, SOX8, GLI3
GOTERM_MF_FAT	GO:0008227~amine receptor activity	3	0,017851256	HRH3, HTR4, HTR5A
GOTERM_BP_FAT	GO:0009891~positive regulation of biosynthetic process	9	0,019294201	THRB, GDF7, SOX5, KITLG, PDGFC, PRDM16, ALX4, SOX8, GLI3
GOTERM_BP_FAT	GO:0008283~cell proliferation	7	0,019313594	CCKBR, ERBB2, GBX2, KITLG, COL8A1, CXCL12, RUNX3
GOTERM_CC_FAT	GO:0005576~extracellular region	17	0,020432577	FGF19, GDF7, FAM20A, ERBB2, KITLG, CXCL12, TIMP3, CPZ, RSPO1, HTRA1, PGLYRP2, LASS1, WNT9B, PDGFC, GPX7, COL8A1, IGFBP3
GOTERM_BP_FAT	GO:0006355~regulation of transcription, DNA-dependent	16	0,021716587	IRX3, ZNF264, THRB, SOX14, SOX5, ZNF232, PRDM16, SOX8, DLX5, HIF3A, GBX2, TFAP2A, ALX4, KCNH2, RUNX3, FOXE3
GOTERM_CC_FAT	GO:0043005~neuron projection	6	0,021894608	ARHGEF7, MAP1B, CACNA1G, DPYSL5, CACNA1H, HCN4
GOTERM_BP_FAT	GO:0035295~tube development	5	0,022386494	RET, GBX2, TFAP2A, CXCL12, GLI3
UP_SEQ_FEATURE	compositionally biased region:Poly-His	3	0,022785563	CACNA1G, CACNA1H, CECR6
INTERPRO	IPR000910:High mobility group, HMG1/HMG2	3	0,024361302	SOX14, SOX5, SOX8
SP_PIR_KEYWORDS	voltage-gated channel	4	0,02548687	CACNA1G, CACNA1H, HCN4, KCNH2
GOTERM_BP_FAT	GO:0030001~metal ion transport	7	0,025547166	ACCN4, CACNA1G, CACNA1H, SLC30A2, HCN4, KCNH2, ABCC8
GOTERM_BP_FAT	GO:0019935~cyclic-nucleotide-mediated signaling	4	0,02557719	HRH3, ADCY5, HTR4, HTR5A
UP_SEQ_FEATURE	compositionally biased region:Poly-Ala	6	0,025720883	IRX3, HRH3, LASS1, GBX2, CECR6, FOXE3
GOTERM_BP_FAT	GO:0051252~regulation of RNA metabolic process	16	0,026045843	IRX3, ZNF264, THRB, SOX14, SOX5, ZNF232, PRDM16, SOX8, DLX5, HIF3A, GBX2, TFAP2A, ALX4, KCNH2, RUNX3, FOXE3
GOTERM_BP_FAT	GO:0051956~negative regulation of amino acid transport	2	0,028923178	HRH3, SLC15A1
GOTERM_BP_FAT	GO:0048704~embryonic skeletal system morphogenesis	3	0,031498918	TFAP2A, ALX4, GLI3
GOTERM_BP_FAT	GO:0006873~cellular ion homeostasis	6	0,035569163	HRH3, CCKBR, ERBB2, CACNA1G, KCNH2, CXCL12
SMART	SM00398:HMG	3	0,03682104	SOX14, SOX5, SOX8
GOTERM_MF_FAT	GO:0022843~voltage-gated cation channel activity	4	0,037317825	CACNA1G, CACNA1H, HCN4, KCNH2
GOTERM_BP_FAT	GO:0055082~cellular chemical homeostasis	6	0,0376921	HRH3, CCKBR, ERBB2, CACNA1G, KCNH2, CXCL12
UP_SEQ_FEATURE	compositionally biased region:Poly-Arg	4	0,038294931	GBX2, CACNA1H, BSN, FOXE3
GOTERM_BP_FAT	GO:0032332~positive regulation of chondrocyte differentiation	2	0,038379918	THRB, GLI3
GOTERM_BP_FAT	GO:0051953~negative regulation of amine transport	2	0,038379918	HRH3, SLC15A1
GOTERM_BP_FAT	GO:0006936~muscle contraction	4	0,0386893	CACNA1G, CACNA1H, HCN4, KCNH2
GOTERM_BP_FAT	GO:0045449~regulation of transcription	20	0,039128664	IRX3, MTERF, ZNF264, THRB, GDF7, SOX14, SOX5, ZNF232, PRDM16, SOX8, GLI3, DLX5, ZNF681, HIF3A, GBX2, TFAP2A, ALX4, KCNH2, RUNX3, FOXE3
GOTERM_BP_FAT	GO:0048754~branching morphogenesis of a tube	3	0,040047403	GBX2, CXCL12, GLI3
UP_SEQ_FEATURE	domain:EGF-like 3	3	0,040336425	ASTN2, MEGF11, MEGF10
GOTERM_CC_FAT	GO:0005615~extracellular space	8	0,040504106	RSPO1, GDF7, HTRA1, LASS1, KITLG, PDGFC, IGFBP3, CXCL12
GOTERM_BP_FAT	GO:0042981~regulation of apoptosis	9	0,041032521	SMO, ARHGEF7, ERBB2, ALX4, IGFBP3, TIMP3, GLI3, RUNX3, DAPK1
GOTERM_CC_FAT	GO:0030425~dendrite	4	0,041066113	MAP1B, CACNA1G, DPYSL5, CACNA1H
BIOCARTA	h_shhPathway:Sonic Hedgehog (Shh) Pathway	2	0,041104281	SMO, GLI3
GOTERM_BP_FAT	GO:0007389~pattern specification process	5	0,041253852	SMO, GBX2, ALX4, CXCL12, GLI3
UP_SEQ_FEATURE	site:Calcium ion selectivity and permeability	2	0,041589994	CACNA1G, CACNA1H
GOTERM_BP_FAT	GO:0060562~epithelial tube morphogenesis	3	0,042307444	RET, TFAP2A, GLI3
GOTERM_BP_FAT	GO:0043067~regulation of programmed cell death	9	0,04309474	SMO, ARHGEF7, ERBB2, ALX4, IGFBP3, TIMP3, GLI3, RUNX3, DAPK1
PIR_SUPERFAMILY	PIRSF037272:bone morphogenetic protein	2	0,043517507	GDF7, LASS1
KEGG_PATHWAY	hsa05217:Basal cell carcinoma	3	0,043729773	SMO, WNT9B, GLI3
GOTERM_BP_FAT	GO:0010941~regulation of cell death	9	0,043885699	SMO, ARHGEF7, ERBB2, ALX4, IGFBP3, TIMP3, GLI3, RUNX3, DAPK1
GOTERM_BP_FAT	GO:0031099~regeneration	3	0,044614401	SMO, MAP1B, TIMP3
KEGG_PATHWAY	hsa04340:Hedgehog signaling pathway	3	0,045180078	SMO, WNT9B, GLI3
SP_PIR_KEYWORDS	ionic channel	5	0,045281674	ACCN4, CACNA1G, CACNA1H, HCN4, KCNH2
GOTERM_BP_FAT	GO:0050767~regulation of neurogenesis	4	0,047353835	SMO, IRX3, MAP1B, SOX5
GOTERM_BP_FAT	GO:0021513~spinal cord dorsal/ventral patterning	2	0,047745946	SMO, GLI3
GOTERM_BP_FAT	GO:0051955~regulation of amino acid transport	2	0,047745946	HRH3, SLC15A1
GOTERM_BP_FAT	GO:0032891~negative regulation of organic acid transport	2	0,047745946	HRH3, SLC15A1
GOTERM_BP_FAT	GO:0003012~muscle system process	4	0,048765241	CACNA1G, CACNA1H, HCN4, KCNH2
GOTERM_MF_FAT	GO:0005261~cation channel activity	5	0,048789731	ACCN4, CACNA1G, CACNA1H, HCN4, KCNH2
GOTERM_BP_FAT	GO:0050801~ion homeostasis	6	0,049045365	HRH3, CCKBR, ERBB2, CACNA1G, KCNH2, CXCL12
UP_SEQ_FEATURE	domain:EGF-like 12	2	0,049700621	MEGF11, MEGF10
GOTERM_BP_FAT	GO:0001763~morphogenesis of a branching structure	3	0,050579893	GBX2, CXCL12, GLI3
GOTERM_BP_FAT	GO:0048469~cell maturation	3	0,05180588	RET, SOX8, RUNX3
GOTERM_BP_FAT	GO:0001932~regulation of protein amino acid phosphorylation	4	0,052383691	GDF7, KITLG, PDGFC, IGFBP3
INTERPRO	IPR003938:Potassium channel, voltage-dependent, EAG/ELK/ERG	2	0,053409413	HCN4, KCNH2
UP_SEQ_FEATURE	domain:EGF-like 2	3	0,053729812	ASTN2, MEGF11, MEGF10
UP_SEQ_FEATURE	domain:EGF-like 11	2	0,053730475	MEGF11, MEGF10
GOTERM_BP_FAT	GO:0048706~embryonic skeletal system development	3	0,054289695	TFAP2A, ALX4, GLI3
SP_PIR_KEYWORDS	DNA binding	5	0,055349791	THRB, GBX2, SOX5, TFAP2A, GLI3
GOTERM_BP_FAT	GO:0010604~positive regulation of macromolecule metabolic process	9	0,055991487	THRB, GDF7, SOX5, KITLG, PDGFC, PRDM16, ALX4, SOX8, GLI3
GOTERM_BP_FAT	GO:0045941~positive regulation of transcription	7	0,056505453	THRB, GDF7, SOX5, PRDM16, ALX4, SOX8, GLI3
GOTERM_BP_FAT	GO:0021511~spinal cord patterning	2	0,057022119	SMO, GLI3
GOTERM_BP_FAT	GO:0031102~neuron projection regeneration	2	0,057022119	SMO, MAP1B

GOTERM_BP_FAT	GO:0048729~tissue morphogenesis	4	0,057662483	RET, TFAP2A, GLI3, RUNX3
UP_SEQ_FEATURE	domain:EGF-like 14	2	0,057743451	MEGF11, MEGF10
GOTERM_BP_FAT	GO:0055085~transmembrane transport	7	0,058493123	CACNA1G, CACNA1H, SLC30A2, UCP1, HCN4, KCNH2, ABCC8
GOTERM_BP_FAT	GO:0006874~cellular calcium ion homeostasis	4	0,059999708	HRH3, CCKBR, CACNA1G, CXCL12
GOTERM_BP_FAT	GO:0007166~cell surface receptor linked signal transduction	15	0,061411681	RET, CCKBR, GDF7, ERBB2, ADCY5, HTR4, CXCL12, GLI3, CPZ, SMO, RSPO1, HRH3, WNT9B, PDGFC, HTR5A
GOTERM_BP_FAT	GO:0008045~motor axon guidance	2	0,061626776	ERBB2, CXCL12
GOTERM_MF_FAT	GO:0004993~serotonin receptor activity	2	0,063193785	HTR4, HTR5A
GOTERM_BP_FAT	GO:0010628~positive regulation of gene expression	7	0,063435163	THRB, GDF7, SOX5, PRDM16, ALX4, SOX8, GLI3
GOTERM_MF_FAT	GO:0003677~DNA binding	18	0,063594105	IRX3, MTERF, ZNF264, THRB, SOX14, SOX5, ZNF232, PRDM16, SOX8, GLI3, DLX5, ZNF681, HIF3A, GBX2, TFAP2A, ALX4, RUNX3, FOXE3
GOTERM_CC_FAT	GO:0043209~myelin sheath	2	0,063780894	HRH3, ERBB2
GOTERM_BP_FAT	GO:0055074~calcium ion homeostasis	4	0,063993274	HRH3, CCKBR, CACNA1G, CXCL12
SP_PIR_KEYWORDS	Growth factor binding	2	0,065315125	HTRA1, IGFBP3
UP_SEQ_FEATURE	domain:EGF-like 10	2	0,065719044	MEGF11, MEGF10
UP_SEQ_FEATURE	domain:EGF-like 13	2	0,065719044	MEGF11, MEGF10
GOTERM_BP_FAT	GO:0032330~regulation of chondrocyte differentiation	2	0,066209287	THRB, GLI3
INTERPRO	IPR011489:EMI domain	2	0,066315744	MEGF11, MEGF10
GOTERM_BP_FAT	GO:0051960~regulation of nervous system development	4	0,067275239	SMO, IRX3, MAP1B, SOX5
GOTERM_BP_FAT	GO:0035113~embryonic appendage morphogenesis	3	0,067312548	DLX5, ALX4, GLI3
GOTERM_BP_FAT	GO:0048589~developmental growth	3	0,067312548	SMO, TIMP3, GLI3
GOTERM_BP_FAT	GO:0030326~embryonic limb morphogenesis	3	0,067312548	DLX5, ALX4, GLI3
SP_PIR_KEYWORDS	receptor	12	0,067452435	SMO, RET, TNFRSF10C, HRH3, THRB, CCKBR, UNC5A, ERBB2, HTR4, ABCC8, LRP3, HTR5A
KEGG_PATHWAY	hsa04080:Neuroactive ligand-receptor interaction	5	0,067901428	HRH3, THRB, CCKBR, HTR4, HTR5A
GOTERM_BP_FAT	GO:0015672~monovalent inorganic cation transport	5	0,069513328	ACCN4, UCP1, HCN4, KCNH2, ABCC8
UP_SEQ_FEATURE	domain:EMI	2	0,0696818	MEGF11, MEGF10
GOTERM_CC_FAT	GO:0016021~integral to membrane	32	0,070230564	CYB5R1, CLSTN2, SLC15A1, ERBB2, ADCY5, CABP7, KITLG, ASTN2, MEGF11, MEGF10, HRH3, UNC5A, HS6ST3, SLC30A2, HCN4, HTR5A, RET, ST6GAL2, CCKBR, HTR4, PCDH9, UCP1, B3GAT2, SMO, TNFRSF10C, ACCN4, LASS1, CACNA1G, CACNA1H, KCNH2, ABCC8, LRP3
GOTERM_BP_FAT	GO:0006875~cellular metal ion homeostasis	4	0,070633381	HRH3, CCKBR, CACNA1G, CXCL12
GOTERM_BP_FAT	GO:0003002~regionalization	4	0,071484679	SMO, GBX2, ALX4, GLI3
SP_PIR_KEYWORDS	Proto-oncogene	4	0,073108059	SMO, RET, THRB, ERBB2
GOTERM_MF_FAT	GO:0022832~voltage-gated channel activity	4	0,074126036	CACNA1G, CACNA1H, HCN4, KCNH2
GOTERM_MF_FAT	GO:0005244~voltage-gated ion channel activity	4	0,074126036	CACNA1G, CACNA1H, HCN4, KCNH2
GOTERM_BP_FAT	GO:0019725~cellular homeostasis	6	0,076713719	HRH3, CCKBR, ERBB2, CACNA1G, KCNH2, CXCL12
GOTERM_BP_FAT	GO:0042325~regulation of phosphorylation	6	0,076713719	GDF7, ADCY5, ERBB2, KITLG, PDGFC, IGFBP3
GOTERM_BP_FAT	GO:0055065~metal ion homeostasis	4	0,078461435	HRH3, CCKBR, CACNA1G, CXCL12
GOTERM_BP_FAT	GO:0006350~transcription	16	0,078590127	MTERF, ZNF264, THRB, SOX14, SOX5, ZNF232, PRDM16, SOX8, GLI3, ZNF681, GBX2, HIF3A, TFAP2A, ALX4, RUNX3, FOXE3
INTERPRO	IPR006212:Furin-like repeat	2	0,079048387	RSPO1, ERBB2
GOTERM_BP_FAT	GO:0006811~ion transport	8	0,079509923	ACCN4, CACNA1G, CACNA1H, SLC30A2, UCP1, HCN4, KCNH2, ABCC8
GOTERM_BP_FAT	GO:0042307~positive regulation of protein import into nucleus	2	0,079824989	SMO, GLI3
GOTERM_BP_FAT	GO:0030512~negative regulation of transforming growth factor beta receptor signaling pathway	2	0,079824989	HTRA1, PRDM16
GOTERM_BP_FAT	GO:0032890~regulation of organic acid transport	2	0,079824989	HRH3, SLC15A1
UP_SEQ_FEATURE	domain:IGFBP N-terminal	2	0,081470735	HTRA1, IGFBP3
SP_PIR_KEYWORDS	extracellular matrix	4	0,081499943	WNT9B, COL8A1, TIMP3, CPZ
SP_PIR_KEYWORDS	Homeobox	4	0,08228288	IRX3, DLX5, GBX2, ALX4
GOTERM_BP_FAT	GO:0035108~limb morphogenesis	3	0,084140441	DLX5, ALX4, GLI3
GOTERM_BP_FAT	GO:0035107~appendage morphogenesis	3	0,084140441	DLX5, ALX4, GLI3
UP_SEQ_FEATURE	domain:TSP type-1	2	0,085367497	RSPO1, UNC5A
GOTERM_BP_FAT	GO:0002009~morphogenesis of an epithelium	3	0,087059513	RET, TFAP2A, GLI3
INTERPRO	IPR002126:Cadherin	3	0,087146558	RET, CLSTN2, PCDH9
INTERPRO	IPR000867:Insulin-like growth factor-binding protein, IGFBP	2	0,087441463	HTRA1, IGFBP3
INTERPRO	IPR000024:Frizzled cysteine-rich domain	2	0,087441463	SMO, CPZ
GOTERM_BP_FAT	GO:0019220~regulation of phosphate metabolic process	6	0,087503969	GDF7, ADCY5, ERBB2, KITLG, PDGFC, IGFBP3
GOTERM_BP_FAT	GO:0051174~regulation of phosphorus metabolic process	6	0,087503969	GDF7, ADCY5, ERBB2, KITLG, PDGFC, IGFBP3
GOTERM_BP_FAT	GO:0007507~heart development	4	0,087586879	FGF19, SMO, ERBB2, GLI3
GOTERM_BP_FAT	GO:0007224~smoothened signaling pathway	2	0,088793305	SMO, GLI3
GOTERM_BP_FAT	GO:0021545~cranial nerve development	2	0,088793305	SMO, GLI3
INTERPRO	IPR017970:Homeobox, conserved site	4	0,089637729	IRX3, DLX5, GBX2, ALX4
GOTERM_BP_FAT	GO:0048736~appendage development	3	0,090008941	DLX5, ALX4, GLI3
GOTERM_BP_FAT	GO:0060173~limb development	3	0,090008941	DLX5, ALX4, GLI3
UP_SEQ_FEATURE	domain:EGF-like 1	3	0,092009394	ASTN2, MEGF11, MEGF10
INTERPRO	IPR001356:Homeobox	4	0,092301117	IRX3, DLX5, GBX2, ALX4
UP_SEQ_FEATURE	domain:EGF-like 9	2	0,093112103	MEGF11, MEGF10
UP_SEQ_FEATURE	domain:FZ	2	0,093112103	SMO, CPZ
GOTERM_BP_FAT	GO:0048678~response to axon injury	2	0,093245125	ERBB2, MAP1B
SP_PIR_KEYWORDS	wnt signaling pathway	3	0,093604184	RSPO1, WNT9B, CPZ
INTERPRO	IPR012287:Homeodomain-related	4	0,094997406	IRX3, DLX5, GBX2, ALX4
GOTERM_BP_FAT	GO:0045860~positive regulation of protein kinase activity	4	0,095197844	ADCY5, ERBB2, KITLG, PDGFC
SP_PIR_KEYWORDS	ion transport	6	0,096668725	ACCN4, CACNA1G, CACNA1H, SLC30A2, HCN4, KCNH2
GOTERM_MF_FAT	GO:0019838~growth factor binding	3	0,097111557	HTRA1, ERBB2, IGFBP3
GOTERM_BP_FAT	GO:0001569~patterning of blood vessels	2	0,097675523	GBX2, CXCL12
GOTERM_BP_FAT	GO:0021532~neural tube patterning	2	0,097675523	SMO, GBX2
UP_SEQ_FEATURE	compositionally biased region:Poly-Pro	5	0,098302557	HRH3, GBX2, KCNH2, LRP3, CECR6
SMART	SM00261:FU	2	0,098313722	RSPO1, ERBB2
GOTERM_CC_FAT	GO:0005891~voltage-gated calcium channel complex	2	0,098412963	CACNA1G, CACNA1H
GOTERM_BP_FAT	GO:0060429~epithelium development	4	0,099103068	RET, DLX5, TFAP2A, GLI3
GOTERM_BP_FAT	GO:0030005~cellular di-, tri-valent inorganic cation homeostasis	4	0,099103068	HRH3, CCKBR, CACNA1G, CXCL12
